# A Critical Comparison of the Advanced Extraction Techniques Applied to Obtain Health-Promoting Compounds from Seaweeds

**DOI:** 10.3390/md20110677

**Published:** 2022-10-28

**Authors:** Eva Quitério, Clara Grosso, Ricardo Ferraz, Cristina Delerue-Matos, Cristina Soares

**Affiliations:** 1 Ciências Químicas e das Biomoléculas/CISA, Escola Superior de Saúde—Instituto Politécnico do Porto, Rua Doutor António Bernardino de Almeida, 400, 4200-072 Porto, Portugal; 2 LAQV-REQUIMTE, Instituto Superior de Engenharia do Porto, Instituto Politécnico do Porto, Rua Doutor António Bernardino de Almeida 431, 4249-015 Porto, Portugal; 3 LAQV-REQUIMTE, Departamento de Química e Bioquímica Faculdade de Ciências, Universidade do Porto, R. do Campo Alegre, 4169-007 Porto, Portugal

**Keywords:** bioactive compounds, conventional extraction, enzyme-assisted extraction, microwave-assisted extraction, pressurised-liquid extraction, supercritical-fluid extraction, ultrasound-assisted extraction, ultrasound-microwave-assisted extraction

## Abstract

Marine macroalgae are rich in bioactive compounds that can be applied in several fields, mainly food, cosmetics, and medicine. The health-promoting effects of bioactive compounds, such as polyphenols, polysaccharides, carotenoids, proteins, and fatty acids, have been increasingly explored, especially regarding their antioxidant activity and improvement in human health. To extract these valuable compounds, advanced technologies that include Supercritical-Fluid Extraction (SFE), Pressurised-Liquid Extraction (PLE), Ultrasound-Assisted Extraction (UAE), Microwave-Assisted Extraction (MAE), Enzyme-Assisted Extraction (EAE), Ultrasound-Microwave-Assisted Extraction (UMAE) and Liquefied Gas Extraction (LGE) have been assessed due to their notable advantages over the conventional methods (Solid–Liquid and Soxhlet extraction). These advanced techniques are considerably influenced by different extraction parameters such as temperature, pressure, type of solvent, extraction time, solvent:solid material ratio, power (MAE, UAE, and UMAE), enzymes used (EAE), and factors related to the macroalgae matrix itself. Optimizing these process parameters for each method is critical to obtain better efficiency results for the targeted bioactive compounds. Macroalgae are natural sources with undeniable beneficial effects on human health. In this context, optimising the extraction techniques discussed in this review should prioritise exploiting these valuable resources’ wide range of bioactive properties.

## 1. Introduction

Little-explored organisms are found in the marine environment, representing approximately half of global biodiversity [1]. Seaweeds show particular interest, mainly due to the medicinal properties derived from their distinct nutritional compounds [2]. Macroalgae or seaweed are found worldwide in marine habitats [3]. They are divided into three taxonomic groups, namely brown algae (Phaeophyta), green algae (Chlorophyta) and red algae (Rhodophyta) [4], depending on their chemical structure and the presence of pigments such as phycobilins for red, chlorophyll for green, and fucoxanthin for brown algae [5].

The most valuable cultivated seaweeds are *Euchema*, *Laminaria* spp., *Gracilaria* spp., *Undaria pinnatifida* (Harvey) Suringar, *Kappaphycus* spp., and *Porphyra* spp. [6], produced for various purposes and applications.

Historically, seaweed compounds have been used as gelling, thickening, and emulsifying agents in various foodstuffs. However, seaweeds are now considered a source of health-promoting compounds that depend on growing conditions such as water temperature, salinity, nutrients, and light [7].

Seaweeds are a rich source of bioactive compounds and secondary metabolites such as bioactive peptides, lectins, carotenoids, polysaccharides, fatty acids, flavonoids, proteins, tocopherols, and phytosterols, with potential application in food, cosmetic and pharmacological industries, being notably distinct from terrestrial plants [2]. Due to these biocompounds, macroalgae present a wide variety of bioactivities such as antioxidant, anti-viral, anti-fungal, antibacterial, antiproliferative, anti-inflammatory, neuroprotective, adipogenesis, and antidiabetic [8].

Seaweeds are a natural photosynthetic resource, and they are characterised by pigments, such as chlorophyll and carotenoids. The latter is responsible for providing, in addition to the antioxidant activity, the antimicrobial, antiproliferative and antihypertensive activities, mainly derived from the major carotenoid, fucoxanthin, evidencing a potential power in combating diseases induced by oxidative stress [4]. In addition to these compounds, algae contain a relatively higher amount of unsaturated fatty acids when compared to saturated ones exhibiting acetylcholinesterase (AChE) inhibition, potentially promoting protection against neurodegenerative disorders [4]. At the same time, high fibres content due to the presence of non-digestible polysaccharides in their cell wall, such as fucoidan and laminarin, have been shown to have anti-viral effects, anti-tumour, anti-inflammatory, hypoglycaemic and also antioxidant activity [4]. Furthermore, seaweeds produce bioactive compounds such as phlorotannins to protect them against UV radiation, stress, and herbivores [7].

As a result of the wide variability of classes of target biocompounds, and their physical and chemical properties, it is essential to find the most efficient methods for extracting these bioactive compounds subsequently, to optimise the extraction protocol. Furthermore, yields and extract composition are influenced by the extraction conditions. It has been reported that factors such as the applied solvent, the solid:liquid ratio, the extraction time and temperature impact these parameters [9].

The most frequently employed method is solid-liquid extraction using suitable solvents such as hexane, methanol, chloroform, ethanol, acetone, and water [7,9,10,11]. The use of traditional solvents for the extraction process of bioactive compounds reveals several disadvantages, such as the need for large amounts of organic polluting solvents, the often-time-consuming extraction, selectivity problems, and co-extraction of unwanted interfering materials [9]. Plus, using toxic solvents like methanol and chloroform to extract bioactive compounds implies some restrictions on their application concerning food and pharmaceutical products, contrary to water and ethanol extractions [12]. To this end, a search for the optimization of this technique culminated in developing several more efficient, fast, and environmentally friendly techniques for extracting bioactive compounds from natural sources.

Besides healthy biocompounds, macroalgae can accumulate undesirable elements naturally present in marine environments, such as iodine and toxic elements (As, Cd, Hg, and Pb) [3,13]. For this reason, there are considerable safety concerns related to seaweed consumption due to potential health adverse events associated with exposure to these heavy metals [14]. Furthermore, these elements and iodine levels vary between algae species, seasons, and harvest locations [3]. Therefore, regularly consuming seaweeds may lead to the risk of toxicity in humans and high iodine status, which can negatively affect thyroid function [15,16]. Consequently, extracting bioactive compounds instead of directly consuming algae may prevent excessive ingestion of potentially toxic elements [14].

In turn, the extraction of these critical natural compounds from algae can be carried out by various approaches ranging from conventional methods such as Soxhlet extraction, hydrodistillation, maceration with alcohol and traditional solid-liquid extraction [7,9,11] to new advanced alternative methods, the latter of which today more than ever are based on the concept of green and eco-friendly techniques [4].

Moreover, the sustainability of the methods applied to extract and purify the bioactive compounds is fundamental. Presently, extraction techniques’ efficiency and quality are sought, but developing environment-friendly extraction processes is preferred over conventional extraction techniques [17]. These alternative methods rely on the principles of green chemistry, resulting in innovation through the selection of renewable resources, the use of alternative and reduced quantities of solvents (mainly water), and as a consequence of the automation of processes, the reduction of energy consumption [17]. With the combination of these requirements, these methods show a better selectivity for isolating the desired compounds. Simultaneously, the formation of by-products and unwanted reactions during the extraction is avoided while allowing shorter extraction time and efficient performance at lower temperatures [4].

Moreover, these techniques may be scalable and provide advantages over conventional extraction methods while coupled to other processes within a biorefinery approach [18]. Biorefinery concepts are particularly interesting nowadays since efforts to minimise or eliminate industrial waste are increasing, culminating in the need for greener extraction techniques. This approach relies on applying a sequential extraction process without manipulating the biomass while effectively recovering diverse compounds. Furthermore, this procedure shows how a complete biorefinery of by-products can be obtained using the same system by controlling the extraction parameters and coupling different techniques. Therefore, developing efficient techno-economical systems can be a way to systematically optimise energy, capital, or other benefits [19].

From this development, the extraction techniques based on compressed fluids appear to be those capable of meeting the criteria considered adequate under the mentioned perspective, with emphasis on innovative techniques such as Supercritical Fluid Extraction (SFE), Pressurised Liquid Extraction (PLE), Ultrasound-Assisted Extraction (UAE), Microwave-Assisted Extraction (MAE), Enzyme-Assisted Extraction (EAE), Ultrasonic-Microwave-Assisted Extraction (UMAE) and Liquefied Gas Extraction (LGE).

Several reviews have been published recently reporting the application of advanced extraction techniques to macroalgae metabolites. Dobrinčić et al. [20] reported the advances in the extraction of brown algae polysaccharides such as laminarin, alginate and fucoidan used in functional foods, cosmetics and pharmaceutical products. These authors compared conventional extraction methods, usually complex and time-consuming, with advanced techniques, namely microwave-assisted extraction, ultrasound-assisted extraction, pressurised liquid extraction and enzyme-assisted extraction. Lomartire et al. [21] reviewed the isolation of seaweed polysaccharides with potential applications as therapeutical agents using advanced extraction techniques. The current state-of-art alginates extraction, such as microwave and ultrasound-assisted extractions, and the determination of their physicochemical properties and overall applications focusing on biomedical purposes have been presented [22]. In another review [23], different processing technologies and extraction techniques such as liquid extraction with solvents and advanced techniques like enzyme-assisted extraction, supercritical fluid extraction, and pressurised liquid extraction were discussed regarding their effect on the bioactive compounds and nutritional value of seaweeds and their compounds. Gallego et al. [18] reviewed using compressed fluids, mainly under sub- and supercritical conditions, to extract bioactive compounds from natural matrices, including seaweeds. The extraction of brown seaweeds using supercritical carbon dioxide (SCCO_2_) and subcritical water (SWE) was discussed, focusing on the combination of these technologies together or with other extraction methods while also presenting the challenges regarding scale-up [24]. Wang et al. [25] reviewed the recent developments for edible algae processing, such as drying and sterilization, including ultrasound, infrared radiation, microwave, and radio frequency. Extraction techniques were also discussed, including microwave-assisted (MAE), ultrasound-assisted (UAE) and pulsed electric field-assisted extractions (PEF). Ramos et al. [26] reviewed the use of ionic liquids (ILs), considered environmentally-friendly and green solvents, combined with advanced extraction techniques, such as microwave-assisted (MAE), ultrasound-assisted (UAE) or subcritical water extractions (SWE) to obtain bioactive compounds from several sources such as waste or seaweeds. McReynolds et al. [27] and Mehariya et al. [28] reviewed the recent studies on the use of deep eutectic solvents (DES) and natural deep eutectic solvents (NADES), respectively, for the extraction of value-added biocompounds from different matrices, including micro- and macroalgae using advanced extraction techniques such as SWE, UAE and MAE. NADESs are a new generation of ionic liquids and are constituted by a hydrogen bond acceptor (HBA) and a hydrogen bond donor (HBD). These components form a network of hydrogen bonds that results in a lower melting point of the solid mixtures. NADEs are considered renewable and biodegradable, biocompatible, stable, with high solubilizing power, and, depending on their composition, they exhibit a wide range of polarity [28]. Recently, a significant advantage of using NADES for trace elements co-extraction from *Fucus vesiculosus* Linnaeus was also reported when compared with other solvents such as water and acetone 70% [29].

Therefore, the main objective of this work is to analyse the most innovative green extraction techniques, such as SFE, PLE, UAE, MAE, EAE, UMAE and LGE, applied to recover bioactive compounds from macroalgae. The emphasis was on the most relevant characteristics of each technique and the process parameters’ behaviour to identify the ideal extraction conditions for the maximum efficiency and quality of the desired compounds while comparing them with the most traditional approaches. The seaweeds reported in this review are: *Ahnfeltiopsis pygmaea* (J. Agardh) P.C.Silva & DeCew, *Ascophyllum nodosum* (Linnaeus) Le Jolis, *Codium fragile* (Suringar) Hariot, *Caulerpa racemosa* (Forsskål) J.Agardh, *Ceramium flexuosum* C. Agardh, *Carpophyllum plumosum* (A. Richard) J. Agardh, *Codium tomentosum* Stackhouse, *Chondrus crispus* Stackhouse, *Cladophora herpestica* (Montagne) Kützing, *Chnoospora minima* (Hering) Papenfuss, *Chaetomorpha antennina* (Bory) Kützing, *Cladophora glomerata* (Linnaeus) Kützing, *Ecklonia cava* Kjellman, *Eisenia bicyclis* (Kjellman) Setchell, *Enteromorpha prolifera* (O.F. Müller) J. Agardh, *Ecklonia radiata* (C. Agardh) J. Agardh, *Fucus serratus* Linnaeus, *F. vesiculosus*, *Gracilaria gracilis* (Stackhouse) Steentoft, L.M. Irvine & Farnham, *Gracilaria corticata* (J. Agardh) J. Agardh, *Grateloupia lithophila* Børgesen, *Himanthalia elongata* (Linnaeus) S.F. Gray, *Hormosira banksii* (Turner) Decaisne, *Ishige okamurae* Yendo, *Kappaphycus alvarezii* (Doty) Doty ex Silva, *Laminaria japonica* Areschoug, *Laminaria hyperborea* (Gunnerus) Foslie, *Laminaria digitata* (Hudson) J.V. Lamouroux, *Monostroma nitidum* Wittrock, *Osmundea pinnatifida* (Hudson) Stackhouse, *Pyropia haitanensis* (T.J. Chang & B.F. Zheng) N. Kikuchi & M. Miyata in J.E. Sutherland & al., *Padina commersonii* Bory, *Saccharina japonica* (Areschoug) C.E. Lane, C. Mayes, Druehl & G.W. Saunders, *Sargassum coreanum* J. Agardh, *Sargassum horneri* (Turner) C. Agardh, *Sargassum muticum* (Yendo) Fensholt, *Sargassum polycystum* C.Agardh, *Scytosiphon lomentaria* (Lyngbye) Link, *Sphaerotilus natans* Kützing, *U. pinnatifida*, *Ulva prolifera* O.F. Müller, and *Ulva fasciata* Delile. Furthermore, a critical comparison will be made between these extraction techniques regarding extraction yield, environmental impact, and potential cost.

## 2. Methods

The research was carried out in Google Scholar, Web of Science, PubMed, and editors’ databases.

Search terms were combined in different manners and included: macroalgae, seaweeds, advanced extraction techniques, advanced extraction technologies, green extraction techniques, SFE, PLE, UAE, MAE, EAE, UMAE and LGE.

**Figure 1:**
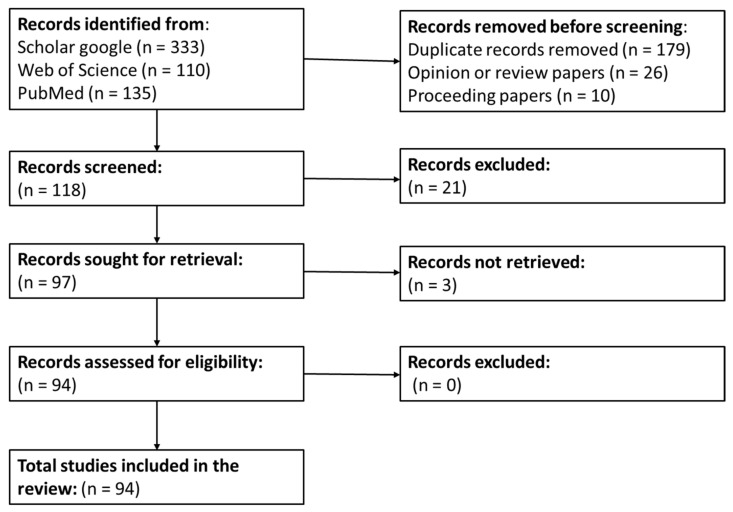
PRISMA flow diagram representing the original papers included in Section 3.

Articles were selected based on two criteria: (1) articles that specifically address the extraction of compounds derived from macroalgae, and (2) only articles written in English were used (Figure 1).

## 3. Extraction Techniques Used to Obtain Health-Promoting Compounds from Seaweeds

The choice of the extraction technique for obtaining a health-promoting fraction from seaweeds is crucial and depends on the research’s goals. The polarity of the extract, the target compound’s thermal stability, the amount of extract, the available equipment, and the potential application must be considered while planning the procedure. Several extraction options are divided into two groups: conventional techniques (solid-liquid, Soxhlet extraction) and advanced techniques (extraction by ultrasound, microwaves, pressurised liquid, supercritical fluid, and enzyme assisted).

### 3.1. Pre-Extraction Sample Preparation

Sample preparation is the first step that usually comprises washing, drying, and grinding of the sample to optimise the extraction efficiency and the safety of the final extract.

Fresh seaweed samples, usually collected from coastal areas or beaches, are usually subject to cleaning steps before extraction procedures. First, seaweeds are washed with filtered seawater and then with distilled water to remove residual sediments, impurities, and salts [30]. After the cleaning step, algal materials are then dried using different techniques: surface-dried with a paper towel, chopped into small fragments and freeze-dried at a temperature of −110 °C [31] to −80 °C for 72 h [10], oven-dried (70 °C, 72 h) [32], dehydrated in a food dehydrator (60 °C) [33], 41 °C [34] or even sun (40 °C) or shade (38 °C) dried for 24 h [33]. Posteriorly, for extraction purposes, seaweed samples are finely ground using a mechanical blender [10] to ensure uniformly distributed mass and a higher surface-to-volume ratio [7].

**Figure 2:**
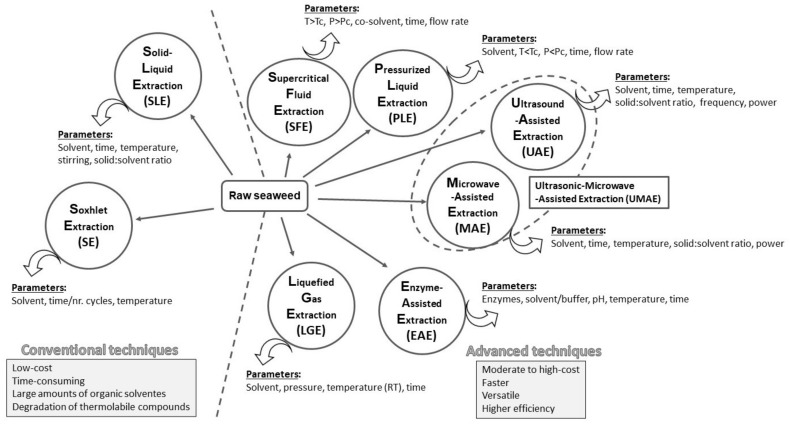
Schematic representation of the extraction techniques discussed in the review.

The dried and ground material is then subjected to different extraction techniques to prepare extracts rich in bioactive compounds. The most common extraction method is solid-liquid extraction (SLE), but more environmentally friendly advanced extraction techniques, namely, SFE, UAE, PLE, MAE, EAE, UMAE and LGE, are also used (Figure 2). The following chapters will discuss a literature review on applying these techniques to seaweeds.

### 3.2. Solid-Liquid Extraction (SLE)

SLE is a technique long applied to extract compounds from different natural sources, characterised by using organic solvents [35,36]. It is based on removing desired soluble constituents from a solid or semisolid matrix using a suitable liquid phase [11]. The process consists of the mixture of the solvent with the natural material for a specific period, sufficient to ensure the migration of the solute from the solid matrix into the solvent [36]. Before this process, drying water-containing materials is recommended to promote increased recoveries of non-polar organic compounds and grounding samples into a powder to increase their surface area [36,37].

Although this is still a widely used technique, SLE involves a time-consuming process using a relatively large quantity of organic solvents considered harmful to the environment [11]. In addition, it is a high-energy technique with mass transfer issues associated with more than one phase in the system [7]. On the other hand, this technique excels in its simplicity and relatively low cost [11]. However, the temperature conditions employed in this method may alter the active molecules, like fucoxanthin, leading to its bioactivity deterioration by the heating process and consequently to low extraction yields [7].

The efficiency of this traditional extraction method is influenced by many correlated factors such as polarity/solubility of both the compounds of interest and the solvent, solvent characteristics, solvent:solid ratio, and sample size and features, among other practical factors like time, temperature, pH and pressure [36,37]. In this sense, the polarity of the solvent should be similar to that of the target analytes, and its solubility must be adequate to extract desired compounds while not affecting the sample matrix [37].

The SLE technique is commonly used for extracting valuable polyphenolic compounds from numerous natural sources with good extraction results [36]. The extraction parameters for the SLE applied to macroalgae are summarised in Table 1.

SLE is the most common extraction method used to extract antioxidant material. This technique was applied to *F. serratus*, *L. digitata*, *G. gracilis*, and *C. fragile*, utilizing different solvents (ethanol:water, methanol:water, hot water, and cold water) to examine the overall extraction efficiency of this technique [38].

As shown in Table 1, the different seaweed species showed significant differences in the extraction yield values. In general, *C. fragile* revealed the highest yields for all the solvents tested, except when methanol:water was employed. The maximum extraction yield was recorded for this green alga (48.2%) using cold water as a solvent, while a minimum value (5.5%) was observed for the red algae *G. gracilis* using hot water [38].

Coldwater extracts presented the higher yield values for most species, followed by methanol extracts, ethanol extracts, and finally hot water extracts, which revealed considerably lower values than the previous ones. These results suggested that the solvent used for the extraction procedure impacted its efficiency. Additionally, indicate that most of the soluble components in seaweeds were of high polarity, which is not unexpected since macroalgae are rich in polysaccharides [38].

The antioxidant capacities of the extracts were evaluated based on the radical scavenging ability, expressed as antiradical power. No significant differences in antioxidant properties were verified between the species for the solvents employed, except for *F. serratus* [38]. The latter revealed the higher radical scavenging power for all solvents tested, while *G. gracilis* presented the minimum antiradical capacity for the four solvents used [38]. These values suggested that extracts from *F. serratus* were the most effective radical scavengers [38]. The solvent used affects the extraction yield and influences the antioxidant activity of the extract. Ethanolic extract from *F. serratus* had the highest radical scavenging activity, while the methanolic extract from *G. gracilis* was the lowest [38]. In general, cold water extracts of all species revealed a more potent antioxidant activity than the other solvents, except for *F. serratus*, in which ethanolic extract showed the highest radical scavenging activity. Contrarily, the methanolic extracts presented the lowest antioxidant activity, excluding for *L. digitata* whose ethanol extract had the lowest antiradical value [38].

For *S. horneri* and *S. japonica* brown algae, SLE was performed using three solvents (hexane, ethanol and acetone: methanol) at a constant temperature (25 °C) and extraction time (1200 min), allowing to determine the influence of the solvent type in the procedure efficiency [10]. It is possible to verify by Table 1 that *S. horneri* presented slightly higher extraction yields for all solvents applied than *S. japonica*. Although fucoxanthin is a dominant carotenoid of brown algae, its content is species-dependent [41]. *S. horneri* presented the maximum extraction yield verified (1.42 ± 0.08 g/100 g) when using hexane as a solvent, while the combination of methanol:acetone for *S. japonica* revealed the lowest value (1.19 ± 0.21 g/100 g) as it is shown in Table 1 [10]. The solvent’s influence on the extraction process’s efficiency was again recognised. The highest yield values were obtained using hexane for both species, followed by ethanol and methanol:acetone extracts. However, higher yield values do not necessarily bring a higher fucoxanthin content because fucoxanthin is a polar compound. It is expected that polar solvents such as methanol and acetone have higher levels of this molecule than non-polar ones such as hexane [42]. The antioxidant activity of the different extracts was assessed based on their hydroxyl radical scavenging activity. Similarly, to the extraction yield, the highest activity values were verified for *S. horneri*, except when methanol mixed with acetone was employed. The hydroxyl radical scavenging activities were proportionately decreased when solvents such as ethanol and hexane were used. Acetone:methanol extracts were the most effective in scavenging H_2_O_2_ for both brown seaweeds, followed by ethanol and hexane extracts. These results corroborate the above mentioned since the greater antioxidant properties are associated with a higher content of bioactive compounds such as fucoxanthin [10].

Relatively to *E. cava* SLE procedure, using different solvents (50% methanol, 100% methanol, and water) allowed to understand its impact on the extraction yield. The water extract showed the higher extraction yield (28.67 ± 1.4%), followed by the 50% methanolic (28.00 ± 1.4%) and 100% methanolic extracts (13.00 ± 1.6%) [39]. This suggests that the use of water as a solvent, even if it is not exclusive, positively affects the efficiency of the process. ROS-scavenging activities of all *E. cava* extracts were analysed using DPPH^•^, expressed as half-maximal inhibitory concentration (IC_50_) values [39]. Differently from before, the 100% methanolic extract exhibited the highest scavenging activity, while the water extract revealed the lowest, suggesting a larger polyphenols content in the methanolic extracts [39].

Natural deep eutectic solvents (NADES) were recently used for the first time to extract phlorotannins from *F. vesiculosus* and *A. nodosum* [40]. Ten NADES solutions composed of choline chloride, lactic acid, betaine, and glucose in various mole ratios were evaluated. Phlorotannins were extracted by maceration for 120 min at 50 °C with a 1:5 raw-material:extractant ratio and evaluated by the Folin–Ciocalteu method. The maximum extraction yield of phlorotannins were 60 and 72% for *F. vesiculosus* and *A. nodosum*, respectively, using NADES solutions prepared with aqueous solution (50–70%) containing choline chloride plus lactic or malic acid or aqueous solution (50–70%) plus malic acid and betaine. The results obtained were comparable to the extraction efficiencies of Me_2_CO and EtOH.

Overall, the efficiency of this extraction technique is closely related to the type of solvent applied, as the higher yields are usually when cold water or mixtures of water: methanol or ethanol are employed. It is also influenced by factors like extraction time and temperature used. Usually, SLE requires extended extraction times due to the large quantities of solid material extracted using this method [38].

The simplicity of its method characterises this conventional technique, making it widely used since ancient times for the extraction of bio-compounds from natural sources [39,43]. However, this method involves a long extraction time, a limited extraction efficiency, and considerable amounts of polluting solvents, potentially leading to sample and environmental contamination and volatilization losses during concentration steps [11,44]. More and more today, these disadvantages stressed the need to resort to alternative methods characterised by being eco-friendly.

### 3.3. Soxhlet Extraction

Soxhlet extraction is an extensively used approach for extracting valuable analytes from solid samples [37]. This technique is advantageous for desirable products with limited solubility in a solvent and the presence of insoluble impurities in that solvent [45]. In this sense, if the compound presents a high solubility in the solvent, it can be separated from the insoluble substance by simple filtration [46].

Soxhlet method consists of the continuous passage of the solvent through the sample matrix by boiling and condensation, resulting in the transference of the compounds of interest from the solid to the solvent if adequate contact time is promoted [36,47]. The extraction is carried out at temperatures below the solvent’s boiling point for extended periods [44]. Since this extraction procedure usually does not require harsh conditions, selecting a suitable solvent is crucial to achieving an appropriate analyte recovery [37]. For this reason, the polarity of the solvent is a critical aspect that can affect extraction efficiency. The solvent selected should be able to dissolve desired analytes, having a similar polarity to these compounds and not the other components of the solid matrix [36,37]. The most used solvents are organic liquids such as ethanol, methanol, acetone, hexane, or ethyl acetate, pure or mixed in water [36].

The solvent choice is a significant parameter that affects the extraction process, alongside other parameters, such as extraction time, temperature, the ratio of the sample, and the properties of the sample intended for extraction [48].

The main disadvantage of this solvent extraction technique is the prolonged and repeated heat that may cause the degradation of thermolabile compounds or result in the formation of artefacts [45,46]. In addition, because of its low selectivity, an evaporation/concentration step is generally necessary after extraction [47,49].

Despite continuing to be a relatively time-consuming process when treating a series of solid samples, it is faster than other conventional methods, less solvent is consumed, and high purity and greater yields are also obtained [37,45]. In addition, this method has easy operation and generally does not require filtration [49].

Soxhlet technique is widely used for many solid biological samples, including the recovery of lipids (fatty acids) and phenolic antioxidants from seaweeds [36,50].

Different extraction parameters and corresponding yields for lipid compounds extracted from different seaweeds using Soxhlet are present in Table 2.

Different extraction yields were verified for *C. glomerata* using three solvents (hexane, acetone, and ethanol). Contrary to what was verified before, when hexane was used as a solvent, a slightly higher extraction yield was obtained than ethanol (Table 2). Acetone revealed the highest extraction efficiency (27.4 ± 0.91%) and ethanol, the lowest yield of fatty acids was verified (24.1 ± 0.87%) [51].

M. Yuvarani et al. [52] extracted lipids also from *C. glomerata* with several solvents (hexane, toluene, isopropanol, methanol, chloroform, chloroform: methanol and hexane: isopropanol) at different extraction times (90, 150 and 210 min). The results suggested the primary role of the extraction solvent and extraction time on the efficiency of the Soxhlet technique. Chloroform:methanol presented the best extraction yields compared to the other solvents for all the extraction times tested, while the lowest values for all extraction times were obtained when chloroform was used. Thus, the hexane:isopropanol mixture also revealed higher extraction yields than the individual solvents, especially isopropanol. These results were verified because when adding a more polar solvent, the strong links between lipids and proteins of polar lipids in the cellular membrane are easily broken, and therefore the lower polarity solvent can reach neutral lipids, and better extraction yields were verified [52]. Regarding the influence of the extraction time, the results showed that the lipid extraction efficiency increases by increasing the time. The extraction time of 210 min revealed the higher yields for all solvents, the highest being verified when chloroform:methanol was applied for the extraction procedure (18%) and the lowest for chloroform (2.3%). This can be because the diffusion of lipids through the cell membrane is slow, mainly when non-polar solvents are used, thus requiring longer extraction times for better results [52].

Halim et al. [53] reported that non-polar solvents interact with the long hydrophobic chains of fatty acids and neutral lipids through van der Waals interactions, promoting the solubilization of these lipids [53]. Contrarily, polar solvents, besides solubilizing non-polar molecules, also solubilise polar lipids related to the cell wall by breaking down the electrostatic interactions and hydrogen bonding [54]. Additionally, the viscosity of ethanol is lower than hexane, so the penetration into the cell wall is easier, promoting higher lipid extraction yields [55]. Hexane revealed a lower extraction yield (4.12 ± 0.06%), while a greater yield was obtained when using EtOH:Hex (1:2) as a solvent (12.05 ± 0.30). As expected, the viscosity of the EtOH:Hex mixtures is lower than the pure hexane, resulting in greater extraction efficiency. According to Johnson and Lusas [56], this combination favours enthalpic and entropic interactions, resulting in a decrease in Gibbs free energy of the system, thereby increasing the solubility and promoting the transport of lipids through the cell wall.

As seen in Table 2, hexane was present in the different Soxhlet procedures analysed, being widely used for this type of extraction because of its stability, low greasy residual effects, boiling point, and corrosiveness [52]. However, it demands more prolonged extraction and solvent consumption and is mainly used with non-polar/neutral lipids [54].

In short, a suitable solvent for lipid extraction should have a high affinity for lipids, low boiling point, low toxicity, low cost, and be easy to recover [57].

Generally, when the solvent polarity increases, the extraction yields also increase. Simultaneously, longer extraction times resulted in better yields (see Table 2). Both parameters revealed a significant influence on the extraction efficiency of the Soxhlet technique.

This conventional method is widely applied due to advantages such as simple methodology, low-cost apparatus, extraction of analytes from a larger sample mass, and the wide range of compounds that can be extracted from different solid matrices [37]. However, it presents some considerable drawbacks, including the longer extraction time, the large amounts of solvent used, and the possible thermal decomposition of the analytes [37], which do not fail to make this a reference technique.

### 3.4. Supercritical Fluid Extraction (SFE)

SFE is a green extraction technology broadly applied to recover valuable non-polar or mid-polar compounds such as lipids, essential oils, and carotenoids [18], both at the laboratory and industrial levels.

This technique is based on using solvents in their supercritical conditions, which means temperature and pressure are raised above their critical point. Under those conditions, supercritical fluids present physicochemical characteristics between gases and liquids, generally acquiring higher density but maintaining similar viscosities and intermediate diffusivities to gas [58].

Since various fluids can be used at supercritical conditions, carbon dioxide is the preferred solvent for the extractions of bioactive compounds from natural sources. Carbon dioxide presents several advantages, including mild critical conditions. It is non-toxic, non-flammable, non-explosive, safe, environmentally friendly, readily available, and cheap [59]. One of the utmost interest properties to be used in the food industry is that, because CO_2_ is a gas under room conditions, it is quickly eliminated from the extracts during decompression, obtaining solvent-free extracts [17]. However, the use of supercritical CO_2_ is limited by its low polarity [18], which means it can only extract non-polar or compounds of low polarity [4]. Although to cope with this issue, small amounts of polar co-solvents such as ethanol or methanol can be used to extract polar compounds [60]. These co-solvents are more polar than CO_2_, therefore expanding the range of compounds achievable by increasing the polarity of the supercritical mixture [17].

Conditions during the extraction, particularly pressure and temperature, affect the selectivity and solubility of the various compounds in the supercritical fluid [61]. Carbon dioxide has a relatively low critical temperature (31.1 °C) and pressure (73.8 bar), which makes it an excellent solvent for extracting heat-sensitive bioactive compounds [62], maintaining them preserved, and no degradative changes can occur. In addition to temperature, pressure, supercritical solvent, proportion and nature of co-solvent, other factors are involved related to the sample and the extraction process, such as water content, particle size, dispersant agent, sample amount, SCF flow rate, mode of extraction, extraction time or fractionation [17].

Supercritical extraction may be conducted in three ways: static, dynamic, and combining both modes. In the static mode, the supercritical fluid circulates and contacts with the sample matrix for a specified time and then is released to the sample trap [63]. On the other hand, for dynamic SFE, the sample is continuously supplied with a fresh supercritical fluid and continuously swept into the collection device. For this reason, increasing the supercritical fluid flow rate may enhance extraction efficiency [63]. In combination extraction mode, first, static extraction is applied for a certain period, followed by the dynamic mode, resulting in improved extraction yields [64].

Consequently, the interaction of the different parameters in optimising a particular process may be very complicated. Therefore, determining the best extraction conditions is imperative to obtain the most effective isolation of bioactive compounds.

SFE has been widely applied to isolate bioactive compounds from natural sources, including marine macroalgae phenolic compounds and carotenoids [4].

Table 3 shows the different operating parameters evaluated in this green method and the bioactive compounds extracted from different macroalgae sources, with the respective extraction yield.

Previous studies have shown that the extraction of fucoxanthin from brown seaweed is very low when using pure SC-CO_2_; however, using a co-solvent shows an increase in the extraction yield [10]. Relatively to *S. horneri* and *S. japonica*, fucoxanthin extraction performed using SC-CO_2_ with ethanol (see Table 3), presented extraction yields of 1.41 ± 0.15 and 1.09 ± 0.56 g/100 g DW, respectively. Although fucoxanthin is the primary carotenoid in several brown seaweed, its content varies significantly with the season and life cycle of the algae, thus influencing the yield values obtained through the extraction method [67,68]. Comparing the antioxidant activity of both extracts by the phosphomolybdenum method, *S. horneri* (38.91 ± 3.79 mg/g α-tocopherol DW) revealed a higher antioxidant effect than that of *S. japonica* (30.74 ± 0.13 mg/g α-tocopherol DW) extracts, which may be directly related to the fact that the former shows a higher extraction yield value. Regarding the antimicrobial activity, the half-maximal inhibitory concentration (IC_50_) value of SC-CO_2_ with ethanol extract from *S. horneri* is higher than *S. japonica* [10]. According to Sivagnanam et al. [10] the activities decreased proportionately when using solvents such as hexane and ethanol since these can have potentially dramatic effects on the chemical species.

For the *U. pinnatifida* various extraction conditions were tested: different temperatures, pressure, and flow rate in a total extraction time of 180 min (see Table 3). When analysing the influence of the temperature, it is noticed that no significant variations were verified in the extraction yield values, except at the temperature of 25 °C, where the total extraction yield obtained (0.53 ± 0.05 g/100 g) was considerably lower when compared with the maximum verified yield (1.22 ± 0.04 g/100 g) obtained at 40 °C and pressure of 40 MPa [65]. However, this yield value does not necessarily translate into a higher fucoxanthin content. Quitain et al. [65] reported that the extracts obtained from liquefied CO_2_ at a lower temperature of 25 °C presented twice the amount of fucoxanthin content, although its extraction yield was the minimum verified. This means that lower temperatures are more selective for the extraction of fucoxanthin rather than other compounds present in the lipid content of *U. pinnatifida*, since the dielectric constant of liquid CO_2_ at 25 °C, when compared with other compounds, may be similar to fucoxanthin [65]. Concerning pressure, different values were tested ranging from 20–40 MPa, being that in this interval, no significant effects were verified in the extraction yield values, as well as when varying the flow rate in the range of 1.0–4.0 mL/min [65]. As already mentioned, the extraction yield does not necessarily directly correlate with the percentage of recovery of the compound of interest. It was verified that the recovery rate of fucoxanthin increases with decreasing temperature, which is most likely due to its increased solubility in SC-CO_2_, high pressure, and high CO_2_ flow rate under supercritical conditions. So, at a temperature of 25 °C as the density of the supercritical CO_2_ is higher, an increased recovery of fucoxanthin is expected, although, at this temperature, the lowest yield was obtained, possibly explained by the lower diffusivity of liquid CO_2_. In addition, high temperatures may also cause fucoxanthin, a heat-sensitive compound, to degrade, affecting the content of the extract [65].

Yields of carotenoids, fucoxanthin, and phlorotannins from *L. japonica* using SC-CO_2_ with various co-solvents (sunflower oil, soybean oil, canola oil, EtOH, H_2_O) under three sets of trial conditions (45 °C/20 MPa, 50 °C/25 MPa, 55 °C/30 MPa) were analysed (see Table 4) [66]. It was possible to notice that after adding any of the researched co-solvents, the yield was significantly improved when compared with the control (SC-CO_2_ only), being the pressure of 30 MPa and temperature of 55 °C the closest to the optimal conditions for most co-solvents, since that was where the highest yield values were obtained [66]. These results can be explained due to the increase in solvent density, associated as already referred to, with a higher temperature and consequent swelling of the matrix, improving the diffusion process and the solubilization of compounds [66]. Among the five co-solvents, sunflower oil revealed the best extractions yields of fucoxanthin (0.49–0.70 mg/g), with significantly higher values than the control (0.19–0.28 mg/g), followed by canola oil (0.44–0.53 mg/g), soybean oil (0.45–0.50 mg/g), and ethanol (0.39–0.43 mg/g). For phlorotannins, the most effective co-solvent in improving the extraction yield was water (0.30–0.61 mg/g), while for carotenoids, all vegetable oils present almost equal extraction yield (1.20–2.20 mg/g) [66]. The effectiveness of sunflower oil compared to the other co-solvent oils may be due to less viscosity and better solubility, the reason why it can be well mixed with SC-CO_2_, improving its efficiency [69]. Additionally, the use of high oleic sunflower oil, rich in monounsaturated fatty acid (MUFA), possibly enhanced the solubility of fucoxanthin and thus promoted greater extraction yields [70]. Saravana et al. [66] also affirm that the low yields of fucoxanthin and carotenoids when using soybean and canola oils is due to a lower content in MUFA, longer chain, and less volatile fatty acids compared to sunflower oil. Water as a co-solvent only presented considerable yields for phlorotannins extraction, which increased with temperature and pressure. Features like water’s higher polarity, ability to increase the density of the fluid mixture, and swelling of the solid particles may have contributed to the higher extraction yield when water was used as a co-solvent [66]. Although the extraction yield of SFE is lower than SLE, as discussed previously, it allows for obtaining important non-polar compounds such as carotenoids from algae [66].

In summary, both the temperature, pressure and flow rate significantly affect the rate of extraction, the reason why they should be considered regarding the procedure’s final goal. In general, the increasing pressure and temperature increase the extraction rate of the compounds of interest [65,66]. A progressive increase in these parameters promotes an easy dissipation of the molecules through the sample matrices, as well as the higher diffusivity and lower surface tension of SC-CO_2_ have a positive effect on the transport performance of the target compound through the matrix and into the solvent, thereby improving the extraction efficiency [65]. Regarding co-solvents, ethanol is widely used due to its efficient and less toxicity [71]. However, some counterparts in this solvent include expensive and cumbersome solvent separation from the extract and co-extraction of some unwanted compounds in the product, such as chlorophylls [65]. Several other co-solvents like methanol, propanol, butanol, and acetone can enhance the extraction yield, despite their presence may also decrease the extraction selectivity [71].

SFE is advantageous due to its possible application for the extraction of thermosensitive compounds, which is often a limitation for other referenced green techniques. In addition, it allows easy recovery of the solvent used, has excellent suitability for extraction of solid/liquid compounds with less volatility, minimises the use of organic solvents, and reduces the extraction time. Nonetheless, imprecise modelling can affect the efficiency of SFE. Major drawbacks of this technique are the impossibility of scalability due to a lack of a molecular model of solutes diffusion into SCF and the limited use of CO_2_ as a supercritical solvent due to its non-polar nature for polar solutes [72].

### 3.5. Pressurised Liquid Extraction (PLE)

PLE is a technique based on pressurised solvents at high temperatures (consistently below their critical points), maintained in the liquid state during extraction [30].

Generally, the temperature and pressure conditions range between 50–200 °C and 3.5–20 MPa, respectively [7]. In general terms, PLE is faster and requires less solvent volume than conventional techniques, such as Soxhlet extraction [73]. In addition, the enhanced mass transfer rate increases the analyte’s solubility and decreases solvent viscosity and surface tension occurring under PLE conditions. As a result, the solvent penetrates the matrix more easily, reaching deeper areas and increasing the surface contact, thus, improving the mass transfer to the solvent and facilitating the extraction rate [17].

Different solvents can be employed while maintaining the general principles and instrumental requirements. When water is used as the extraction solvent, this technique is also called subcritical water extraction (SWE). Water is kept at temperatures higher than its boiling point, using high pressure to maintain its liquid state [9], allowing it to rapidly and efficiently extract the desired compounds from the matrices. Thus, the extraction’s efficiency and selectivity rate depend on the temperature applied during the process [9].

Nevertheless, this technique is inappropriate for thermolabile compounds sensitive to high temperature and pressure [7]. The possibility of automation is a further advantage of PLE since it helps reduce extraction-to-extraction variation, improving reproducibility [17].

Phenolic compounds, typical from macroalgae and other bioactive compounds, have been successfully extracted via PLE [74]. The variable operating conditions, the bioactive compounds extracted, and the correspondent extraction yield are presented in Table 5.

PLE extraction gives significantly higher extraction yields than SFE. As shown in Table 5, *S. muticum* revealed one of the highest yield values (40.1 ± 0.7%) using ethanol:water as a solvent, at a relatively high temperature and low pressure with an extraction time of 20 min [30].

Dinh et al. [31] extracted phenolic compounds from *S. japonica* studying the differences between SWE and SLE methods. These authors also compared SWE with water and IL and observed that TPC, HPLC profile, and antioxidant activity were higher when extracted with both SWE solvents than in SLE. Therefore, water can be a suitable solvent for recovering phenolic compounds with SWE. However, SWE + IL enhanced in extracting phenolics compared to water. The contents of several phenolic compounds in the extracting products were 1.18 to 7.67-fold higher than in SWE at 175 °C. Although the decomposition of vanillic acid in SWE + IL was observed, the antioxidant activity (DPPH^•^, ABTS^•+^, TAC, and FRAP) of the extracts obtained with SWE + IL increased. These authors concluded that IL is probably a potential catalytic agent for SWE during the extraction of bioactive compounds from natural sources. IL was also used as SWE solvent by Gereniu et al. [78] to extract ĸ-carrageenan from the seaweed *K. alvarezii* obtaining yields up to 78.75%. These authors also compared extracts obtained by IL with aqueous SWE and the conventional method. Crude ĸ-carrageenan was characterised by Fourier transform infrared spectroscopy (FTIR), thermal gravimetric analysis (TGA) and X-ray diffraction (XRD), showing a good similarity with a standard. The antioxidant activity of the extract obtained by SWE + IL was slightly lower due to its low sulphate content compared with aqueous SWE and the conventional method. Nevertheless, the authors concluded that the high extraction yield by SWE + IL and the properties of the extracts shows that the inclusion of IL catalyst is a potential extraction method for crude κ-carrageenan.

Soares et al. [34] extracted *C. tomentosum* and *F. vesiculosus* using a multi-step (four step) SWE with a temperature gradient to search for novel neuroprotective compounds. Due to the presence of arsenic and high iodine contents in the first two extraction steps (room–90 and 90–140 °C), only the fractions obtained in steps 3 and 4 were evaluated for biological activity. However, inhibition of five brain enzymes implicated in Alzheimer’s, Parkinson’s, and major depression aetiology and activity against reactive oxygen and nitrogen species were observed for the step 4 fractions for both seaweed species. Moreover, regarding the chemical composition of the fractions, step 1 fractions were the richest in phenolic compounds. Concerning step 4 fraction obtained at higher temperatures (190–250 °C), Maillard reaction, caramelization and thermo-oxidation occurred, and products such as melanoidins are responsible for the antioxidant capacity and the neuroprotective effects. The same SWE conditions described previously [34] (four steps, different temperatures) were applied by Gomes et al. [75] to *H. elongata* and *E. bicyclis*. These authors observed that, although no significant enzyme inhibition was detected, fractions 3 and 4 presented the highest biological activities, namely DPPH^•^, ABTS^•+^, ^•^NO, and O_2_^•−^ scavenging activity, which can also be explained by the presence of Maillard reaction products.

The extraction rate of the four seaweeds (*F. serratus*, *L. digitata*, *G. gracilis*, and *C. fragile*) using PLE with different solvents and temperatures was studied as an indicator of the total extraction efficiency of the technology [38]. Considerable changes in the extraction rate were observed between the different types of seaweeds (see Table 5). *F. serratus* showed the highest extraction yield, while *G. gracilis* presented the lowest values in all the different solvent combinations used for the extraction procedure. It is also possible to understand the solvent, temperature, and pressure impact on these values. For all species investigated, the extraction yields using hot water as a solvent were highest than other solvents, probably due to the higher temperature and pressure used for the extraction. Raising the temperature improves contact of the analytes with the solvent and enhances the extraction through an increased diffusion rate. Moreover, the solubility of analytes, mass transfer, and decreased solvents’ viscosity and surface tension occurs [80]. Even though the pressure is associated only with keeping the solvent liquid at high temperatures, high pressures can help improve PLE efficiency by forcing organic solvents into the matrix pores of the sample [81]. Both ethanol and methanol extracts showed no significant differences in extraction yield from the same species. The antioxidant activity of the crude extracts derived from the PLE method and solvent combinations were expressed as the antiradical power, which is the reciprocal of the IC_50_ [38]. Moderated differences were observed in these values among species for the PLE extracts, especially in *F. serratus*, which presented a significantly higher activity than the other three species. *F. serratus* ethanol extract demonstrated the most effective radical scavenging activity, while methanol extract of *G. gracilis* was about 141 times lower. It is also possible to note that although hot water shows the best extraction yields, these do not necessarily translate into better antioxidant activity results [38].

For the brown algae *H. elongata*, different solvents (ethanol, hexane, and water), with different dielectric constants and, therefore, able to extract bioactives of different polarities, as well as different temperatures (50, 100, 150 and 200 °C) were tested [76]. Different solvents play a role in extraction efficiency, and according to the solvent polarity, different compounds can be found in the extracts. The results demonstrate that higher yields resulted when higher polarity solvents were employed. The best extraction yield values were also obtained using water as a solvent since it presents a higher polarity, followed by ethanol and hexane [76]. As shown in Table 5, the extraction rate increases significantly with the extraction temperature, which can be explained by an increase in the solubility of the sample compounds with temperature and the improved mass transfer from the sample to the pressurised solvent [76]. This fact can be inferred that the main compound in the seaweed’s chemical composition will be a moderately highly polar compound because this will correspond to a decrease in the water dielectric constant at high temperatures. Thus, the TEAC (Trolox Equivalent Antioxidant Capacity) assay was used for antioxidant bioactivity screening. The results showed differences depending on the type of solvent employed. Ethanol extracts presented the best antioxidant capacities, followed by water and hexane. Interestingly, at increasing water temperature, higher antioxidant values were obtained, while for ethanol, the maximum activity was verified at temperatures of 50 and 100 °C), since after passing these temperatures, there is a progressive decrease [76].

PLE extraction procedure for *F. vesiculosus* was performed with different conditions of the parameters that have a significant effect on PLE efficiency, namely solvent type and temperature [77]. Five different solvents (hexane, ethanol, ethyl acetate, acetone, and ethanol:water 50:50) were used, and three temperatures (80, 120, and 160 °C). Considerable differences were noted in the yield values for the five solvents used. As mentioned before, these results also revealed that the best extraction yields were obtained for the most polar solvents like ethanol:water, ethanol, and acetone, while the lower yields were for ethyl acetate and hexane. Ethanol:water mixture presented the highest extraction yield (34.85–57.19%), possibly for the reasons already explained previously and hexane with the lowest yield values (2.79–4.49%) [77]. As expected, the yield increased with the extraction temperature used in the PLE system for all solvents, being the maximum yield verified (57.19 ± 2.38%) for the temperature of 160 °C with ethanol:water (50:50). Nevertheless, despite ethanol: water 50:50 showing the highest yield, this solvent is not a proper solvent for fatty acid extraction from algae, being ethyl acetate more efficient for this purpose. Antioxidant capacity was tested using the DPPH^•^ assay revealing that *F. vesiculosus* extracts have a good dose-response activity, although the authors did not specify values for the different extracts obtained [77].

The highest extraction yields were obtained when water was used as a solvent. These results indicate that most of the soluble components in seaweeds were highly polar because water has a higher polarity than the other solvents and can more efficiently extract desirable compounds such as polyphenols and polysaccharides [77]. So, it can be concluded that the nature of the extraction solvent has a profound effect on PLE efficiency. Furthermore, high temperatures also impact the efficiency of the extraction process, usually translating into higher extraction yields [76,77].

Saravana et al. [79] used SWE + DES to extract polysaccharides from *S. japonica*. The optimal extraction conditions were 150 °C, 19.85 bar, 70% water content, and L/S ratio of 36.81 mL/g showing yields for alginate and fucoidan of 28.12% and 14.93%, respectively. In addition, FTIR, TGA, and differential scanning calorimetry (DSC) were used to evaluate the extracted alginate and fucoidan. The authors concluded that this process was clean, fast and effective, improving the extraction yield of polysaccharides from seaweeds.

Notably, there is a preferential use of ethanol and water as extraction solvents since they are not considered potentially hazardous to the environment and human health, thus enabling their use in food processing, unlike solvents such as hexane, acetone, ethyl acetate, and methanol [80].

In summary, PLE efficiency can be influenced by temperature and extraction solvents, pressure, extraction time, flush volume, flow rate, and sample weight and their effects can be independent or interactive [82]. PLE increases the wetting of the molecules matrix by solvent and enhances penetration, reducing the solvent’s viscosity employed at elevated temperatures and pressures, resulting in increased solubility, thus promoting a reduced extraction time and high extraction yields [72]. On the other hand, the requirement of sophisticated and specialised automated equipment, which makes this green technique not as economical as others, and the lower recovery rates of potentially thermosensitive compounds at elevated temperatures are disadvantages that limit the use of this approach [72].

### 3.6. Ultrasound-Assisted Extraction (UAE)

UAE can be used as a pretreatment to SLE by destroying the biomaterial and making the target compounds more available, resulting in increased yields [83].

This alternative technique uses ultrasound waves with a frequency between 20 kHz to 100 kHz, which can cause the creation of bubbles and zones of high and low pressure [4]. Hence, the sudden collapse of bubbles creating cavitation near liquid interfaces, causing the breakdown of particles, which means that mass transfer is increased, and bioactive compounds are released from a biological matrix [7].

Ultrasound equipment can be an ultrasonic bath (indirect sonification) or an ultrasonic probe (direct sonification), with the operating conditions and the way ultrasound waves affect the sample being the main differences between these two [84]. For example, the ultrasonic bath operates at a frequency of 40–50 kHz and power of 50–500 W, while the ultrasonic probe can only work at 20 kHz. Additionally, the samples are immersed in the ultrasonic bath, whereas the ultrasonic probe is inserted into the sample [84].

Compared with other novel extraction techniques, ultrasound equipment is cheaper, has an easier operation, and uses a wide variety of solvents [7]. Moreover, UAE operates with low temperatures, enabling the preservation of thermolabile compounds and preventing complete damage to the structure [85]. Additionally, solvents such as ethanol, distilled water, and methanol with different solid:solvent ratios are used in low quantities, and the extraction time is reduced, which makes UAE a fast, inexpensive method compared to traditional ones [4].

It has been shown that polyphenols and antioxidants are the main compounds extracted from macroalgae using UAE [4].

Table 6 presents the extraction parameters of this technique and the different bioactive compounds extracted, referencing the extraction yield for each condition.

In the extraction study of laminarin from *L. hyperborea* and *A. nodosum*, different solvents were used (water and HCl) [87]. No significant differences were verified in the extraction yields for both species, although the content of laminarin varies with species, and compared with *A. nodosum*, the content of laminin is higher in *L. hyperborea*. In addition, this content also suffers the influence of factors like harvesting season and geographical location [87]. The highest laminarin content was measured in the extract of *L. hyperborea* and *A. nodosum* using 0.1 M HCl as a solvent, 6.240 ± 0.008, and 5.822 ± 0.343%, respectively. These results agree with previous studies that reported that using 0.1 M HCl gives higher extraction yields compared to water [91]. The antioxidant activity of the crude laminarin extract was also determined by the DPPH^•^ free radical scavenging inhibition assay [87]. As before, the type of solvent used significantly impacted antioxidant activity. Notable differences were verified for the DPPH^•^ inhibition between seaweed species and solvents. The acidic solvent extracts for both seaweeds were found to have higher antioxidant activity than those obtained using water, being the *A. nodosum* extracts the most effective for scavenging DPPH^•^ radicals [87].

Relatively to *E. cava* the influence of different solvents (water, 50% methanol, and 100% methanol) and two extraction times (360 and 720 min) were tested for the UAE procedure [39]. Considerable variances were observed for the different solvents in the extraction yield values, although these are not significant in comparison with the different times used for each solvent extraction [39]. As observed in Table 6, the best extraction yields were obtained when using water as a solvent, followed by 50% methanol and 100% methanol. It is also possible to verify that the extraction yields increased as the ultrasound extraction time increased for all three solvents. The water extract with 720 min showed the highest extraction yield of 34.33%, and the 100% methanol extract revealed the lowest value of 16.00 ± 1.4% for the time of 360 min [39]. These results indicate that the extraction yield is highly time dependent. ROS-scavenging activities of all *E. cava* extracts obtained by ultrasound extraction were tested using DPPH^•^ [39].

ROS scavenging activity is a simple and fast-related method that allows for the measurement of the capacity of the compounds to act as free radical scavengers and, in this way, to evaluate the antioxidant activity of the substances [92]. These free radicals suffer a reduction reaction by either the process of hydrogen or electron donation. Therefore, substances that perform this reaction can be considered antioxidants and radical scavengers [93].

It was verified that the methanolic extracts presented higher radical scavenging properties than the water extracts, which can be associated with higher antioxidant activity. Therefore, it was demonstrated that free radical scavenging activity increases with ultrasound extraction time. Among these results, the 100% methanolic extracted for 720 min exhibited the highest scavenging capacity, while the water extract prepared at 360 min showed the lowest activity [39]. Furthermore, since the radical-scavenging activity related to the polyphenols content increases with the increase of polyphenolic content [94], it is possible to suggest that the methanol extracts presented higher activities showing greater effectiveness for the extraction of these compounds than water [39]. These results indicate that the extracts prepared from *E. cava* by ultrasound extraction with methanol as a solvent have good radical scavenging activities.

The extraction yields of the three seaweeds (*S. muticum*, *O. pinnatifida*, and *C. tomentosum*) using water as a solvent are presented in Table 6. *C. tomentosum* revealed the highest extraction yields, followed by *O. pinnatifida* and *S. muticum* (see Table 6). *C. tomentosum* and *O. pinnatifida* show values of extraction yield relatively close, 45–47%, and 47–49%, respectively, while brown algae (*S. muticum*) present almost half of the values verified for the former (25–27%) [88]. This can be explained by the diversity and complexity of polysaccharides in the algal cell wall, which may reduce the extraction efficiency [95].

Obluchinskaya et al. [90] compared the efficacy of UAE using NADES (25 °C, 60 min) with conventional extraction (maceration, 60 °C, 1 h, 700 rpm) in the extraction of phlorotannins, fucoxanthin and ascorbic acid from *F. vesiculosus*. They concluded that UAE allowed an increased recovery for different compounds, 1.1–2.7 folds higher than the conventional one.

Comparing both bath and probe results, although both techniques apply ultrasound to the sample, there were notable differences in the process efficiency, as the probe promoted lower yield values [87]. However, the treatment time was considerably less than ultrasound bath procedures (see Table 6).

The efficiency of these methods is mainly associated with the bubble cavities phenomena created by ultrasound waves, as these effects allow greater solvent penetration into the cellular materials and improve mass transfer [87,88]. In addition, the cavitation bubbles generate macroturbulence, high-velocity interparticle collisions, and perturbations in microporous particles of the biomass, resulting in disruption of biological cell walls, which facilitate the release of bioactive compounds from the biological matrix [87,88].

Generally, the increase in the extraction time promotes better extraction yields in this alternative technique, although it should be noted that factors such as species, location, and harvesting season also affect the content of compounds of interest [39]. Additionally, the solvent applied seems to have a notable impact on these values and, consequently, on the antioxidant activity resulting from the extracts [39,87].

UAE is considered an emerging potential technology for extracting seaweeds and plant compounds. It is a simple technique, is less time-consuming, economically viable, and promotes the reduction of laboratory wastes. However, its use may be compromised since active compounds like carotenoids can be degraded due to oxidative pyrolysis caused by hydroxyl radicals during cavitation. In addition, high ultrasound waves can destroy bioactive molecules, resulting in undesirable changes in extracted components [72].

### 3.7. Microwave-Assisted Extraction (MAE)

MAE is another technique that represents an environmental and economical alternative, offering the possibility of obtaining cheap and high-quality products labelled as “green” according to environmental standards, mainly because of the reduced process time and solvent amount [96].

This method combines microwave and traditional solvent extraction, and its principle is based on ionic conduction and dipole rotation, which act directly on the molecules and co-occur [4]. Microwave heating causes energy absorption by polar molecules losing no heat to the environment while disrupting cells. Destructed cells facilitate faster mass transfer and diffusion out of solid, where mass and heat transfer act synergistically and in the same direction [97]. In microwave extraction, microwave power heats to a controlled temperature samples and solvents enclosed in high-quality vessels [98].

MAE can be performed in two central systems: open or closed vessels. Closed vessels operate at higher temperature and pressure conditions, while open vessels operate at atmospheric pressure. This latter can be more productive and safer, and it is possible to process larger samples [4]. Moreover, process conditions are suitable for thermolabile compounds [99].

This alternative extraction technique offers superior flexibility, such as vessel options, sample size, temperature, pressure, amounts of solvent, and the number of samples making this extraction method attractive for high-throughput sample preparation and routine extraction of natural products [98]. Therefore, it is essential to find MAE’s ideal operating conditions to promote the highest efficiency and quality in the extraction process. Relatively, to extract bioactive compounds from natural sources like plants, this technique ruptures cell walls, favouring the diffusion of thermolabile chemical content from the plant matrix to the solvent [72].

Polyphenols, polysaccharides [4], fucoidans, carotenoids, and minerals [7] are the most microwave-assisted extracted compounds from macroalgae.

The operating parameters that can diverge in this alternative technique are present in Table 7, as well as the bioactive compounds extracted and extraction yield.

Different extraction parameters including extraction time (20, 25, 30 and 35 min), microwave power (450, 500, 550 and 600 W), ratio of solvent to material (20, 25, 30 and 35 mL/g) and ethanol concentration (25, 30, 35 and 40%) were tested for the extraction of polyphenols from *E. prolifera* using MAE [101]. There were some significant differences in yield values obtained due to the combination of the factors relevant to this technique’s efficiency. The solvent choice was considered for its ability to separate target components and absorb microwave energy [101]. As shown in Table 7, the highest extraction yield was obtained when using 30% ethanol for 25 min, while using 35% ethanol showed the lowest yield of polyphenols in 30 min [101]. The irradiation time also seems to influence the extraction yield, as verified in Table 7. The progressive increase in time up to 25 min promotes an increase in yield values, however after this time a decrease is seen in almost all cases, except for the use of 35% ethanol at a power of 550 W. Excessive microwave exposure may cause polyphenols to deteriorate [101], as it has been reported in other studies for flavonoids [106] and triterpenoid saponins [107], and thus cause the decrease seen in the obtained values of extraction yield. Generally, a higher volume of solvent will increase the recovery rate in conventional extraction techniques, but a higher volume of solvent in MAE may result in a lower recovery rate [101]. Luo et al. [101] reported the smallest decrease in polyphenols yield when the solvent volume increased above 25 mL/g. A similar trend was reported by Li et al. [108], who also observed a reduction in phenolic compounds extraction when the solvent volume was increased over 40 mL/g. This may be due to the large volume of ethanol, causing the material to swell excessively in water and absorb the active ingredients [101]. It was found that by increasing the solvent-to-material ratio up to 25 mL/g, the yield also increased, reaching its maximum of 0.912 mg/g, decreasing when the ratio exceeded 25 mL/g [101].

Regarding microwave power on the extraction yield of polyphenols, the extraction efficiency improved when raising the microwave power from 200 to 500 W. There was a progressive decrease from 500 to 700 W. These results demonstrate that excessive microwave power may cause the loss of the bioactive compounds of interest, like polyphenols [101]. This fact is attributed to the increased power of moisture content in the sample that destroyed the biologically active substances [109]. According to A. Delazar et al. [98], applying low microwave power and longer extraction time is preferable to optimise MAE protocol.

For *U. prolifera* the effect of temperature (90, 120, and 150 °C) and acid concentration (0.1, 0.01, and 0.05 M HCl) on the MAE process was examined [103]. Table 7 revealed that higher extraction yields were obtained when using 0.01 M HCl in all temperatures tested, while the lowest values were attained by increasing acid concentration (0.1 M HCl). In turn, the temperature was also revealed to have an impact on the efficiency of this technique. At higher temperatures (150 °C), generally, lower extraction yields were observed, while at a lower temperature (90 °C), the yield values were increased [103]. The highest yield (36.38 ± 0.94%) was obtained at 120 °C with 0.01 M HCl, and the minimum yield (6.09 ± 0.65%) was verified when the extraction condition was 150 °C, 0.1 M HCl [103]. Comparing these two parameters (temperature and acid concentration), acid concentration appears to have a more significant role in polysaccharide yield, with the lowest yields obtained for the highest acid concentration. The acid is probably capable of hydrolysing the polysaccharides, making them unable to be precipitated efficiently, resulting in a low polysaccharide yield [103]. The antioxidant capacities from *U. prolifera* extracts were analysed by the DPPH^•^ radical scavenging assay [103]. The results showed that extraction conditions affect the extract’s inhibitory effects. Considerable differences in radical scavenging activities were verified related to the extraction temperatures and acid concentration. The temperature of 150 °C revealed the highest scavenging effects for all acid concentrations used, while the polysaccharides extracted at 90 °C did not show DPPH^•^ radical scavenging activity. At 120 °C, only the 0.1 M HCl extract presented some antioxidant activity, which is the lowest value compared to the extracts from 150 °C. The highest scavenging effect (27.6%) was observed from polysaccharides extracted at 150 °C, and 0.1 M HCl. As Yang et al. [110] stated, these results may be due to the relationship between the antioxidant activities of polysaccharides and the degree of sulphur content affected by different extraction conditions.

The extraction efficiency in terms of phlorotannin for three brown seaweeds (*C. flexuosum*, *C. plumosum*, and *E. radiata*) was tested using an MAE protocol with water as a solvent, 160 °C, an extraction time of 3 min and a solvent/material ratio of 30 mL/g [104]. It was verified that the extraction yield of phlorotannins was significantly different (Table 7), meaning that the content of this bioactive molecule depends on the seaweed species. *C. flexuosum* presented the highest extraction yield (15.8 ± 0.3%), followed by *C. plumosum* (9.2 ± 0.6%) and *E. radiata* (2.0 ± 0.1%). The recovery of phlorotannins is highly dependent on the extent of macroalgae cell wall degradation since these compounds are present in two central locations within the cell, namely in the cell wall and cytoplasmic organelles (physodes) [104]. Therefore, the results indicate that microwave conditions were sufficiently energetic to partially depolymerise the algal cell wall resulting in the release of cell wall-bound phlorotannins. Although, the apparent low phlorotannin content of *E. radiata* and the high content of *C. flexuosum* makes it difficult to establish a direct relationship between the extraction efficiency and the degree of distortion of the cell wall since the extraction parameters were the same for all species [104]. However, species’ differences should be considered and the impact of factors such as life cycle stage, location, and harvest season [111]. The antioxidant activity of phlorotannins extracted from all three brown algae by MAE was evaluated by FRAP activity and DPPH^•^ free radical scavenging ability. The three species of brown seaweed presented considerable differences in antioxidant activities. *C. flexuosum* showed the highest FRAP activity and DPPH^•^ radical scavenging ability, succeeded by *C. plumosum* and *E. radiata* having the lowest antioxidant activity. According to previous studies, high values of polyphenols are associated with a higher antioxidant activity [112], which suggests that *C. flexuosum* presents a higher content of this class of antioxidant molecules than the other two seaweeds.

In a recent study [105], nine combinations of DES with MAE were evaluated for extracting polysaccharides from *F. vesiculosus*. The maximum yield of polysaccharides obtained was 116.33 mg/g within an extraction time of 35 min, at 168 °C and a solid-liquid ratio of 39 mg/mL. In addition, the antioxidant and anticancer activity in vitro were evaluated, showing that the extracted polysaccharides presented high antioxidant activities and a strong inhibition effect on the growth rate for human cervical cancer HeLa cells. The authors concluded that DES, is a potential environmentally friendly solvent useful to extract polysaccharides from seaweeds.

In summary, microwave power, solvent type, irradiation time, and solvent-to-material ratio are generally considered to be the most critical factors that affect the yield of MAE. Additionally, the extraction process is affected by particle size and the nature of the natural matrix, as the smaller the particle, the larger the area exposed to the mechanical treatment and, therefore, the greater the penetration of microwaves [98].

MAE’s main advantages are the low consumption of organic solvents, rapid extraction procedure, low energy efficiency, low cost, and improved extraction yields that make this technique a viable option compared to traditional techniques [101,113]. However, its use may be limited by compromised function with dried samples [114] and because it requires an additional filtration process after extraction [44]. Additionally, the high temperature involved in this green method makes it not suitable for use with heat-sensitive bioactives like proteins [113].

### 3.8. Enzyme-Assisted Extraction (EAE)

EAE is also considered a green technique that gained attention as a valuable tool to improve the extraction yield of bioactive compounds from seaweeds [115], which has the potential to be combined with other non-conventional extraction techniques efficiently.

The mechanism of enzyme extraction relays on enzymatic hydrolysis processes to help release targeted compounds into the extracting solvent [30] because of its selectivity. Enzyme-assisted extraction mainly depends on the ability of enzymes to hydrolyse the cell wall components disrupting its structural complexity by forming an enzyme-substrate binding complex [116], releasing the cell content into the solution.

Marine algae cell walls consist of chemically complex and heterogeneous biomolecules, which require specific enzymes, such as carbohydrases and proteases, capable of degrading these complex constituents of natural matrices to release the bioactive compounds of interest [7]. Some commonly used enzymes for this application are Arabinase, Cellulase, Amylase, Protease, Glucanase, Viscozyme, Cellucast, Termamyl, Ultraflo, Carragenanase, Agarase, Xylanase, Kojizyme, Neutrase, Alcalase, and Umamizyme [7].

In this extraction technique, operational parameters such as the temperature of the reaction, pH of the system, enzyme concentration, particle size of the substrate, time of extraction [116], type of solvent (water or buffer with appropriate pH) and agitation [11] play an essential role in extraction efficiency. Furthermore, using appropriate enzyme complexes destroys the bonding of unwanted substances. It releases the target compounds in the aqueous media, which in turn enhances the quality of the product [116] as well as the use of optimum conditions (temperature and pH) for enzymes to maximise extraction yield [95].

Besides the reduced time of extraction and volume of solvent used, EAE poses the additional advantages of higher yields by releasing mostly desired bioactive, combats the limitations of water solubility and insolubility for bioactive compounds, shows high catalytic efficiency and preserves the original efficacy of natural compounds [7,116].

Enzyme-assisted extraction is well suited to the extraction of lipids and oils [116], as in including phlorotannins and other phenolic compounds from seaweeds [7].

Some of the most used enzymes, correspondent operation conditions, compounds targeted, and extraction yield are shown in Table 8.

Two different enzymes were applied for polyphenols extraction from *S. muticum*, namely alcalase and viscozyme. Because these enzymes present a different selectivity, different algal components may be found in the extracts [30]. Alcalase is a natural protease found in *Bacillus licheniformis*, whereas viscozyme is an enzymatic preparation composed of carbohydrases, including arabanase, cellulase, β-glucanase, hemicellulase, and xylanase [30]. Each enzyme has its ideal conditions; the appropriate pH and optimum temperature were kept constant while the extraction time was variable to understand its influence on the efficiency of the extraction process [30,117,118]. As can be observed in Table 8, slightly higher yields were obtained using the 4 h treatments compared to 2 h for both enzymes. The highest yield was verified for the viscozyme after 4 h (23.5 ± 0.1%), and the lowest extraction yield value (13.6 ± 1.4%) was obtained for alcalase using the 2 h treatment. The use of viscozyme promoted better extraction yields compared to alcalase for both 2 and 4 h time. In this regard, carbohydrases appear to be more effective than proteases in weakening cell wall structure and consequently improving extraction efficiency [30]. These values also might suggest that using lower pH values in hydrolysis conditions during the treatment with carbohydrases was more effective in releasing bound polyphenols. Curiously, the TEAC assay’s determination of antioxidant capacity did not follow the same trend [30]. No significant quantitative differences were found between alcalase and viscozyme extracts antioxidant activity that was modest in all cases. However, alcalase extracts possessed higher antioxidant activity than the viscozyme extracts. In addition, the influence of the extraction time was also perceived to the extent that the longer the time for extraction of the bioactive molecules, the lowest the antioxidant activity [30]. This demonstrates that even if the extraction yield was a little different between the two enzymes used, the specificity of the enzymes may or may not favour the release of compounds with an impact on the total antioxidant activity of the extracts [30].

The enzymatic assisted extraction was performed in *C. fragile* and *C. crispus* with different enzymes (cellulase, β-glucanase, ultaflo, and neutrase) at a temperature of 50 °C with an extraction time of 3 h. As reported by Kulshreshtha et al. [115], the cell wall of *C. crispus* is composed of polysaccharides and gelling agents that can interfere with the extraction process. For this reason, the additional use of β-glucanase and ultraflo aimed to improve the extraction of bioactives from *C. crispus*. The results indicated that *C. crispus* generally has a higher extraction yield for all compounds of interest in all enzymes used than *C. fragile* [115].

Concerning the extraction of proteins, as shown in Table 8, higher yields were verified for *C. crispus*, the highest value obtained with the cellulase enzyme (7.1 ± 0.3%) and the lowest values with β-glucanase (4.1 ± 0.4%). On the other hand, *C. fragile* presented considerably lower yield values than the previous ones, with the highest yield obtained using neutrase (2.9 ± 0.1%). Overall, the three carbohydrases were the most effective enzymes for protein recovery [115].

For neutral sugars, both enzymes applied in *C. fragile* revealed similar extraction values; however, for *C. crispus* a significant difference between the maximum yield and minimum yield values was verified. For *C. fragile*, 5.4 ± 0.3% was the highest yield obtained using neutrase, while for *C. crispus* the highest yield was obtained when ultraflo was employed (21.9 ± 0.4%). Generally, the neutral sugar content extracted by carbohydrases was the highest [115]. Relatively to uronic acid, *C. crispus* showed the maximum extraction yield of 1.4 ± 0.0% when using ultraflo and lowest value of 0.8 ± 0.0% with neutrase, while *C. fragile* obtained the highest yield of 0.23 ± 0.0% with cellulase hydrolysis. Once again, carbohydrases showed greater efficiency in the extraction procedure than proteases [115]. Finally, the analysis of the effect of enzyme treatments on the extraction of the sulphated groups demonstrated that a higher percentage yield of sulphates was found in *C. crispus* as compared to *C. fragile* [115]. The latter showed no differences in the extraction yield values for neutrase (0.5% ± 0.0%) and cellulase (0.5% ± 0.0%) [115]. *C. crispus* enzymatic extracts presented significantly higher extraction yields, with the maximum value of 11.7 ± 0.1% for cellulase and a minimum of 8.0 ± 0.1% for β-glucanase [115]. Because some red seaweeds are rich in sulphated polysaccharides [115], *C. crispus* is expected to have a higher yield of sulphates than *C. fragile*, and this fact is confirmed by the extraction yield obtained.

The anti-viral activity was tested against the Herpes simplex virus Type 1 using Vero cell lines [115]. Maximum inhibition of viral activity was observed in the enzymatic extract of *C. fragile* with the use of neutrase (36.5 ± 10.3 μg/mL). In *C. crispus*, the extracts obtained with neutrase exhibited an effective anti-viral effect (77.6 ± 9.6 μg/mL) [115]. No anti-HSV-1 activity was observed in *C. crispus* hydrolysates obtained from enzymatic extraction with cellulase and β- glucanase [115].

According to Fernando et al. [118], EAE was also applied to different seaweeds with different enzymes at the optimum conditions. As shown in Table 9, *G. lithophila* revealed the highest extraction yields for all enzymes applied compared to the other seaweeds, except when using ultraflo, while *C. minima* presented the lowest extraction yields for all enzymes. The maximum yield was 40.0 ± 0.9% for *G. lithophila* when celluclast was employed, while the lower extraction yield obtained was 6.5 ± 0.7% for *C. minima* with ultraflo. When celluclast was applied for the extraction procedure, the highest yield values were verified for all seaweed species. These results suggested that celluclast was more effective in breaking cell walls, resulting in improved extraction yields of organic compounds [118].

Antioxidant activities of the extracts were evaluated by DPPH^•^ scavenging activity. In general, extracts of *S. polycystum* demonstrated higher values of DPPH^•^ scavenging capacity than all other macroalgae species [118]. Contrarily, *U. fasciata* and *C. herpestica* extracts showed a lower DPPH^•^ activity for all enzymes [118]. Furthermore, celluclast extract of *S. polycystum* contained the strongest radical scavenging activity, while ultraflo extract of *U. fasciata* revealed the weakest antioxidant activity [118]. Overall, the celluclast extracts exhibited better antioxidant properties than other enzymatic extracts.

In short, increasing the treatment time translates into an increase in the extraction yield since greater damage can be caused in the cell wall. Additionally, it is worthwhile to understand that optimal operating conditions of the enzymes are required for the best extraction efficiency [116].

In summary, the functioning of this technique is based on the ability of enzymes to hydrolyse the algal cell wall, an important step in releasing active molecules. This is due to the cell wall’s composition, which is constituted mainly of molecules such as polysaccharides that limit the accessibility to bioactive compounds, reducing the extraction efficiency in traditional methods [30]. By that, the better extraction yields verified for carbohydrases suggested that these enzymes are more efficient in breaking the cell wall complex. It should also be noted that given the fact that this technique is solvent-free, the extracts obtained can be easily applied in the cosmetics and food industries [115].

EAE has been widely applied for natural compound extraction because it is an eco-friendly, solvent-free, and cost-effective method [115]. However, some disadvantages may condition its use, including the usually slow process [11] and difficulty in maintaining optimum treatment time and temperature conditions for enzymes [7].

### 3.9. Ultrasonic-Microwave-Assisted Extraction (UMAE)

This technology combines ultrasound and microwave-assisted extraction, coupling both methods’ advantages [119].

UMAE associates ultrasonic cavitation with a high-energy effect of microwaves under low temperature and atmospheric pressure [109]. In this way, the complementary nature of these techniques is notable in that microwaves generate a fast and high thermal energy; however, it presents mass transfer limitations that are overcome with ultrasound, which promotes an intense mixture by cavitation of natural plant compounds with convenient heating [119].

Consequently, combining ultrasonic with microwave could potentially minimise or prevent the degradation of extract, obtain higher yields, extract thermally labile active compounds [109], accelerate the extracting process, and this may improve the extraction of bioactive compounds [120].

This advanced complimentary technique has been effectively applied to extract polysaccharides from different natural materials [121].

Table 10 presents the extraction parameters that can vary within this technique, along with the bioactive compounds obtained and extraction yield.

The combination of ultrasound and microwave for the extraction of polysaccharides from *P. haitanensis*, evaluated the independent and interactive impacts of two variables on the extraction yield, including extraction time (20, 30, and 40 min) and temperature (70, 80 and 90 °C) [122]. The temperature of 70 °C showed a more constant extraction yield for the various extraction times tested than 80 and 90 °C (Table 10). The maximum yield value verified was 19.15% for the temperature of 80°C and extraction time of 30 min, while the lowest was obtained (9.50%) at 90 °C after 20 min. As the results in Table 10 show, the extraction time notably affects the yield values. The increase in time up to 30 min showed a linear increase in extraction yield. However, when exceeded, a decrease was observed in these values. This fact proves, as verified for the techniques applied individually, that a long time of microwave and ultrasound radiation can damage the structure of bioactive molecules, causing decreases in yields [109]. However, the degradation of polar chemicals like phenolic compounds has been reported due to the effect of sono-generated radicals in conjunction with the rapid thermal effect of microwaves [111]. However, a short reaction time might be inadequate for bioactive substances since the longer the irradiation time, the greater the microwave energy from the sample cell. Therefore, the destruction of the cell wall is enhanced, so the yields of the active substances increased obviously [109]. Overall, the temperature of 80 °C revealed better extraction yields, followed by 70 °C and 90 °C. These results suggested that the temperature also has an influential role in the efficiency of the process, resulting in an increase up to 80 °C and after this temperature a decrease. Increased sample temperature may also induce cell rupture, improving extraction efficiency, although it may play a role in the deterioration of active molecules [119].

In summary, the increase in temperature and irradiation time seems to positively impact the extraction yield until the optimum values are reached, after which a decrease is verified. Additionally, the microwave-ultrasound irradiation power is a critical parameter that influences the yield of bioactive compounds insofar as it leads to the rupture of the cell wall, offering a quicker transfer of energy to the solvent and matrix and thus allowing the dissolution of components to be extracted [119].

The UMAE process accounts for some superiorities, including higher extraction efficiency, shorter extraction time, energy savings, and fewer by-products, making this method increasingly popular [122]. In addition, it has the potential to replace traditional hydrothermal extraction methods due to the rapid damage it creates on the seaweed cell wall. However, it is expected to have a higher instrument cost since it uses two techniques in simultaneous [43], and a decline in power with time [42], which can affect the procedure’s efficiency.

### 3.10. Liquefied Gas Extraction

Liquefied Gas Extraction (LGE) is an extraction process that can be performed at room temperature and low pressure (200–1000 kPa), with a low energy consumption, and reduced traces of residual solvent in the biomass. In this sense, raw materials and extract quality are preserved. This technique can be performed using batch or semi-continuous mode [123]. The semi-continuous modes proved to be more adaptable to maintaining the continuously evaporated and recycled gasified solvents until extraction was completed.

The major components of LGE equipment are an extractor, a needle valve to control the solvent flow rate, and an extract storage tank connected in series. Liquefied solvent supply tank can be maintained in a water bath, keeping a saturated vapour pressure [124]. The extraction processes can be performed in isobaric and non-isobaric modes [123]. In non-isobaric conditions, the liquefied gas flows through the raw material using a circulating pump, is evaporated by expansion and liquefied by a compressor. Therefore, precise control of the flow rate and working pressure is mandatory. Additionally, a maximum solid/liquid contact can be established by sending the solvent “up-flow”.

Constant pressure keeps the solvent recirculating without a pump or compressor in isobaric conditions. The system is kept at liquid/vapour equilibrium, maintaining the operating pressure as the solvent vapour pressure. Gravity helps transfer the liquefied solvent from one vessel to another, evaporating in the boiler under the same pressure and finally, the vapours rise and condense, allowing for solvent regeneration. Because no mechanical equipment is used, lower energy consumption and maintenance cost is observed [123].

The most common gases used to perform this type of extraction are n-propane, n-butane and dimethyl ether [123]. Liquefied dimethyl ether (DME) (Tc = 126.85 °C; Pc = 5.37 MPa), in particular, has been employed to extract carotenoids and lipids from different matrices, such as, microalgae [125,126] and macroalgae [124,127,128] but also from plants [129]. DME possess several attractive characteristics which make it an ideal extraction solvent: (1) its normal boiling point is low (−24.8 °C) and, therefore, the final extracts are solvent-free; (2) it has a high affinity for oily substances and partial miscibility with water, allowing the extraction of water and oily substances from wet biomaterials, thus eliminating the high energy consumption process of drying; (3) has been approved as a safe extraction solvent by several agencies, such as the European Food Safety Authority (EFSA), the Food Standards Australia New Zealand, and by the United States; (4) and DME exhibits resistance to autoxidation, unlike other alkyl ethers [128].

In Table 11, are presented the solvents, extraction parameters and bioactive compounds extracted using LGE.

Kanda et al. [124] compared the performance of liquefied DME with SFE-CO_2_ and with Soxhlet extraction using ethanol and concluded that the amount of fucoxanthin extracted from *U. pinnatifida* was higher for liquefied DME extraction. The same authors obtained similar results in another study, where they compared liquefied DME with chloroform–methanol extraction for lutein recovery (0.30 mg/g DW and 0.24 mg/g DW, respectively) [127], showing that DME increases the extraction of this carotenoid.

## 4. Critical Comparison between Extraction Techniques

The extraction techniques presented in the previous chapters have different operating parameters and can be employed regarding different target compounds and bioactivities. The extraction process is the first important step in the screening process of the extracts that affects the active constituents present in the sample qualitatively and quantitatively [130].

In addition to the extraction yield, the appropriate choice and efficiency of the technique consider other parameters such as cost, environmental impact, extraction time, the volume of solvent used, selectivity, and the possibility of scale-up [11]. Table 12 presents a brief comparison between the different techniques highlighting the main advantages and disadvantages.

The previously discussed techniques present different characteristics, and the choice will depend on the targeted compound. All allow the extraction of polyphenols being SLE and UAE, which present the highest yields for these compounds and MAE the lowest. Carotenoids with important health benefits were reported to be extracted by SFE, fatty acids by PLE, and proteins by EAE. EAE also appears to be the technique that allows the extraction of a greater variety of compounds. Soxhlet extraction was only used to extract lipids (fatty acids), with relatively good extraction efficiency. SFE is the method with lower extraction efficiency, contrary to the PLE technique.

Traditional extraction methods like SLE are known for their time-consuming and non-eco-friendly procedure because of the use of high amounts of organic solvents [7], the low yield of bioactive compounds, and significant energy consumption [48]. However, these conventional methods allow the use of large amounts of biomass and are cheaper than alternative methods since the implementation of high-pressure equipment dramatically increases the costs associated with PLE and SFE [11,48].

Soxhlet is advantageous over other conventional methods firstly because it establishes a transfer equilibrium due to the repeated contact between the fresh solvent and the plant material, secondly because of the maintenance of high temperatures in the container of extraction and finally no need for filtration after the process of extraction [131].

Novel extraction techniques like SFE, PLE, MAE, UAE, EAE, and UMAE, in addition to being more environmentally friendly technologies, with a shorter extraction time, can significantly improve extraction efficiency [7].

PLE and SFE are green pressurised methods suitable to extract thermolabile compounds since they can be carried out at low temperatures. In addition, a higher selectivity is verified because they allow obtaining different extracts using distinct pressure and temperature conditions [11]. However, as mentioned above, one of the main disadvantages associated with these techniques is the expensive equipment [48], as well as the fact that for the extraction of polar compounds, the use of toxic modifiers is required, and high-temperature extractions may lead to degradation of thermolabile compounds [11]. Comparing these two techniques, SFE is the procedure of choice to selectively extract bioactive compounds as it is more flexible in solvent choice [18]. However, this technology can be more time-consuming than other alternative techniques and present lower extraction yields [11].

A common pattern of techniques like SLE, PLE, and SFE is high temperatures, which may provoke the targeted bioactive compounds to react during extraction by chemical reactions like Maillard and caramelization reactions [9]. The first reaction occurs effectively at temperatures above 50 °C and is favoured at pH 4–7, while caramelization occurs when specific compounds are heated above their melting points (>120 °C) under acidic (pH 3) or alkaline (pH 9) conditions in the absence of nitrogen-containing components [132].

Maillard reactions mainly contribute to flavour and aroma via non-enzymatic browning and caramelization reactions in food processing and cooking [132]. As a result of these reactions, which occur mainly in the presence of proteins and reducing sugars in natural samples, there is the possible formation of antioxidant compounds [17]. However, this fact raises questions about the potential impact on the overall antioxidant activity of the extracts obtained.

UAE and MAE processes are two advanced methods of extraction based on the cell wall’s disruption, thus making them an easy way of extraction with high efficiency and short treatment time and solvent consumption [11,130]. Additionally, UAE is a cheaper and more suitable technique to extract thermolabile compounds, which results in less destruction of the active constituent since elevated temperatures are not used [4,11,130]. Nonetheless, MAE is associated with higher energy consumption, possible thermal degradation of the molecules when using open vessels and requires only solvents with high dielectric properties [11]. In turn, UAE may damage the quality of extracts due to excess sonication and generate extra heat, which is a considerable limitation for its application [11].

In comparison, MAE is preferred over the UAE because MAE increases the mass transfer through the solid matrix of the sample material and speeds up the mixing of the extraction solvent, thereby maintaining the maximum possible driving forces and ensuring the highest quantity, quality and purity of the active constituents in the extract [10,130]. In general, the simultaneous application of ultrasounds and microwaves by UMAE reveals better extraction yields than these isolated techniques [111].

Comparing modern extraction technologies like UAE, SFE, and MAE to Soxhlet, the former can enhance the kinetics of compound extraction and improve compound recovery [44].

Relatively to EAE, using enzymes like proteases and carbohydrases can increase the recovery of bioactive compounds from seaweeds since they can degrade cell walls and release compounds [7]. More specifically, EAE with proteases can improve the extraction efficiency of fatty acids on account of the ability of these enzymes to hydrolyse the structural proteins in which fat globules are embedded [133]. EAE application can be maximised. However, if the usually slow hydrolyse process, which may take hours to days, high cost, difficulty in maintaining ideal enzyme conditions and unavailability of substrate-specific enzymes are overcome [7,11].

EAE and MAE offer considerable green benefits for industrial and large-scale applications, including reduced extraction time, minimization of solvent use, low energy consumption, and increased yield [7,44]. Similarly, UAE has also been used at the industrial level to extract highly efficient bioactive compounds from natural sources [7].

Investing in cold extraction techniques such as LGE [123] is also a possible alternative for extracting thermolabile biomolecules such as carotenoids from natural sources. The main advantage of the cold solvent extraction process is the retention of the bioactive molecules naturally present in the seaweeds, increasing the extraction yield while reducing energy consumption. Furthermore, as high temperatures are not used, modifications in the natural composition of the seaweeds, including destruction or alterations in the natural structure of numerous bioactive compounds, are not expected. Cold extraction methods are, therefore, preferable for the extraction of thermolabile compounds [134].

Besides using advanced and environmentally friendly extraction techniques, “green solvents” have generated significant interest and growing demand. Water can be regarded as the ultimate greener solvent. It is inexpensive, environmentally friendly, non-toxic and non-flammable. Water is employed for different processes, such as maceration, decoction, infusion, and percolation, but it is not a suitable solvent for non-polar or semi-polar compounds [123]. However, the high polarity of water at higher temperatures and pressures is significantly reduced, allowing for better extraction of non-polar compounds, although compromising the thermolabile ones [123].

Furthermore, the necessity of reducing energy, water, solvent consumption, and carbon emission increases the clamour for environmentally-friendly systems [135]. Therefore, the replacement of the traditional extracting solvents for ionic liquids (IL), deep eutectic solvents (DES) and natural deep eutectic solvents (NADES) is showing significant interest. Overall, these suitable solvents are environmentally friendly and less toxic than conventional extraction solvents and show a high ability to extract a high range of bio-compounds [136]. ILs environmentally friendless is related to their low vapour pressure, non-inflammability and high stability [123,136]. However, the long reaction time at high temperatures for its synthesis [136] and their low biocompatibility and biodegradability [123] are questioning their “greenness”. Compared with ILs and conventional solvents, DES are regarded as “green solvents” because of their biodegradability and lower bacterial toxicity [136].

Natural sources of DESs have replaced synthetic compounds originating Natural Deep Eutectic Solvents (NADESs) [123]. NADES are synthesised by natural, inexpensive, abundant, and recyclable components and therefore are considered non-toxic with no adverse effects. NADES are compatible with food, pharmaceutical, and cosmetic products. Up to date, all NADES evaluated are considered green biodegradable solvents. Therefore, NADES are less toxic and more biodegradable than DES and IL [136].

In conclusion, using these emerging solvents for the extraction process is promising. These solvents potentially have low or no toxicity besides their high solvation ability and versatility [136]. Notably, NADES are allowed in food and pharmaceutical formulations; however, some DES and IL require further investigation. Therefore, the extraction mechanisms involved must be elucidated appropriately to expand the applications of these emerging solvents to recover bioactive compounds [136].

## 5. Conclusions and Prospects

The incorporation of seaweeds or extracts, especially in the food, cosmetic, and pharmaceutical industries, presents a promising potential for developing functional products that positively influence human health.

Nevertheless, seaweed consumers must be aware of the possible presence of toxic contaminants such as heavy metals and excessive iodine content, presenting potential risks to human health.

For this reason, extracting components from seaweeds can avoid these health-related problems while concentrating many novel bioactive components with potential activities against several human diseases.

Conventional extraction techniques, like SLE, are often time-consuming and use large amounts of organic solvents, making them eco-unfriendly, which creates the need to develop novel extraction technologies that can produce extracts of higher value while fighting these impacting problems.

The advanced alternative green extraction techniques presented in this review revealed a tremendous potential for isolating bioactive compounds from marine macroalgae. In addition, they tend to be faster, sustainable, eco-friendly, reproducible, and overall, more efficient than traditional methods.

Compressed fluids-based extraction techniques such as SFE and PLE allow by changing the pressure and temperature conditions, or by altering the solvent in PLE or adding a co-solvent in SFE, the selective extraction of a determined class of compounds to the detriment of others. One of the biggest advantages of these techniques is related to the fact that they reduce the consumption of organic solvents in the case of PLE, or as in SFE, the extracts can be solvent-free, avoiding the toxic effects of these solvents.

Moreover, cell wall disruption techniques, including mechanical (MAE, UAE, and UMAE) and enzymatic (EAE) technologies, can greatly improve extraction yields and reduce extraction time. However, the efficiency of the mechanical methods proved to be strongly affected by the treatment time, increasing with increasing this parameter, thus, the risk of degradation of thermolabile components is also raised. Simultaneously, the solvent:seaweed ratio, temperature, and power contributed to the extraction rate. The EAE is affected by the specificity of the enzymes used and their optimum conditions as well as by the time of treatment, which translates into an increase in extraction yield as it increases.

In addition to considering all these influential parameters, choosing the most appropriate and efficient extraction technique must also consider the nature of plant matrices and the analyte of interest.

The type of extraction technique used, as well as the variation of extraction parameters and factors directly related to the various species of algae, revealed a significant impact on the antioxidant properties of the extracts. Even so, using radical scavenging assays to assess antioxidant activities revealed that seaweed extracts have multifunctional properties, which is a major advantage for food and cosmetical applications.

However, the greatest benefits of these novel green technologies can be achieved only by overcoming limitations such as high-cost and process robustness. Therefore, to scale up these novel technologies, removing technical barriers in the designs and processes should be a priority to exploit the improved extraction of bioactives from algae at an industrial level.

Although alternative extraction techniques described in this review privilege aspects notably more advantageous than traditional techniques, the latter still dominate most laboratories’ daily practices worldwide.

To combat this trend and thus make the use of more sustainable techniques preferable, future approaches to these extraction techniques may be based on the use of alternative green solvents, like ILs, DES and NADES, to replace some of the hazardous solvents still used. The undeniable advantages of these solvents, namely NADES, make them promising economically viable and environmentally friendly alternatives for different extraction applications to supply safe extracts for the food, pharmaceutical and cosmetics industries. Besides, DES and NADES are suitable solvents for several advanced techniques such as PLE, MAE and UAE.

Moreover, these extraction methods can be combined with other processes within a biorefinery approach and provide attractive advantages over conventional extraction protocols, especially in the minimization of wastes related to agri-food products. The simultaneous use of high-pressure extractions and hydrolysis with or without enzymatic assistance, as well as the combination of ultrasound and microwave, have been tested and may promote the development of efficient technical and economic systems.

Cold extraction techniques are also a viable alternative for the extraction of biomolecules from natural sources, preferable regarding the extraction of thermolabile compounds since hot temperatures are not applied in the procedure.

Macroalgae are natural sources with undeniable beneficial effects on human health. In this context, the optimization of the extraction techniques mentioned in the present review should be a priority for better exploiting the wide range of bioactive properties of these valuable resources.

## Figures and Tables

**Table 1:** Solid-Liquid Extraction for bioactive compounds from macroalgae, extraction parameters, bioactive compounds targeted, and extraction yield.

Macroalgae	Extraction Parameters			Bioactive Compounds	Bioactivity	Extraction Yield (%)	Reference
	Solvent (*v*/*v*)	Temperature (°C)	Extraction Time (min)				
*S. horneri* *S. japonica*	Hexane	25	1200	Fucoxanthin	Antioxidant	1.42 ± 0.08 ^a^	[10]
						1.24 ± 0.06 ^a^	
	Ethanol					1.36 ± 0.14 ^a^	
						1.22 ± 0.12 ^a^	
	Acetone:methanol					1.29 ± 0.05 ^a^	
						1.19 ± 0.21 ^a^	
*F. serratus* *L. digitata* *G. gracilis* *C. fragile*	EtOH:Water (80:20)	Room temp.	1440	Polyphenols	---	24.9	[38]
						35.2	
						25.8	
						46.1	
	Methanol:Water (70:30)	Room temp.				26.3	
						36.5	
						29.2	
						34.0	
	Hot water	60				6.2	
						7.9	
						5.5	
						8.8	
	Cold water	Room temp.				35.9	
						35.9	
						25.9	
						48.2	
*E. cava*	50% Methanol	Room temp.	1440	Polyphenols	Antioxidant	28.00 ± 1.4	[39]
	100% Methanol					13.00 ± 1.6	
	H_2_O					28.67 ± 1.4	
*F. vesiculosus* *A. nodosum*	NADES	50	240	Phlorotannins	---	60	[40]
						72	

a: g/100 g DW.

**Table 2:** Soxhlet Extraction for bioactive compounds from macroalgae, extraction parameters, bioactive compounds targeted, and extraction yield.

Macroalgae	Extraction Parameters			Bioactive Compounds	Bioactivity	Extraction Yield (%)	Reference
	Solvent (*v*/*v*)	Temperature (°C)	Extraction Time (min)				
*C. glomerata*	Hexane	---	180	Fatty acids	Antioxidant	24.5 ± 0.78	[51]
	Acetone					27.4 ± 0.91	
	Ethanol					24.1 ± 0.87	
*C. glomerata*	Hexane	69	90	Lipids	---	5.0 ^a^	[52]
			150			6.9 ^a^	
			210			11.76	
	Toluene	111	90			3.7 ^a^	
			150			5.1 ^a^	
			210			9.8	
	Isopropanol	82	90			4.2 ^a^	
			150			6.8 ^a^	
			210			9	
	Methanol	64.7	90			3.0 ^a^	
			150			4.4 ^a^	
			210			8.6	
	Chloroform	61	90			2.3 ^a^	
			150			3.8 ^a^	
			210			7.6	
	Chloroform: MeOH	---	90			6.9 ^a^	
			150			9.5 ^a^	
			210			18	
	Hex: Isopropanol	---	90			5.5 ^a^	
			150			8.1 ^a^	
			210			14.9	

a: Approximated values.

**Table 3:** Supercritical-Fluid Extraction for bioactive compounds from macroalgae, extraction parameters, bioactive compounds targeted, and extraction yield.

Macroalgae	Extraction Parameters					Bioactive Compounds	Bioactivity	Extraction Yield (%)	Reference
	Solvent (*v*/*v*)	Temperature (°C)	Pressure (MPa)	Extraction Time (min)	CO_2_ Flow Rate				
*S. japonica*	CO_2_:EtOH	45	25	120	27 g/min	Fucoxanthin	Antioxidant	1.09 ± 0.56 ^a^	[10]
*S. horneri*							Antimicrobial	1.41 ± 0.15 ^a^	
*U. pinnatifida*	CO_2_	25	40	180	2 mL/min	Fucoxanthin	---	0.53 ± 0.05	[65]
		40	40					1.22 ± 0.04	
		40	20					1.08 ± 0.02	
		40	30					1.06 ± 0.03	
		50	40					1.07 ± 0.01	
		60	40					1.04 ± 0.01	
*L. japonica*	Sunflower oil, soybean oil, canola oil, EtOH, H_2_O	45	20	240	---	Carotenoids	Antioxidant	See Table 4	[66]
		50	25			Fucoxanthin			
		55	30			Phlorotannins			

a: g/100 g DW.

**Table 4:** Supercritical-Fluid Extraction for carotenoids, fucoxanthin, and phlorotannins from macroalgae *L. japonica* and respective extraction yields.

Extraction Yield [mg/g]						
Compounds	SC-CO_2_	Sunflower Oil	Soybean Oil	Canola Oil	EtOH	H_2_O
Carotenoids	0.85 ^a^	1.20 ^a^	1.20 ^a^	1.30 ^a^	1.10 ^a^	0.20 ^a^
	1.00 ^b^	1.60 ^b^	1.50 ^b^	1.60 ^b^	1.20 ^b^	0.25 ^b^
	0.98 ^c^	2.20 ^c^	2.00 ^c^	2.2 ^c^	1.10 ^c^	0.25 ^c^
Fucoxanthin	0.19 ^a^	0.49 ^a^	0.45 ^a^	0.44 ^a^	0.39 ^a^	0.01 ^a^
	0.25 ^b^	0.60 ^b^	0.48 ^b^	0.50 ^b^	0.41 ^b^	0.02 ^b^
	0.28	0.70	0.50	0.53	0.43	0.03 ^c^
Phlorotannins	0.20 ^a^	0.75 ^a^	0.59 ^a^	0.70 ^a^	0.24 ^a^	0.30 ^a^
	0.25 ^b^	---	---	---	0.31 ^b^	0.50 ^b^
	0.29 ^c^	0.15 ^c^	0.10 ^c^	0.13 ^c^	0.35 ^c^	0.61 ^c^

a: 45 °C/20 MPa; b: 50 °C/25 MPa; c: 55 °C/30 MPa.

**Table 5:** Pressurised-Liquid Extraction for bioactive compounds from macroalgae, extraction parameters, bioactive compounds targeted, and extraction yield.

Macroalgae	Extraction Parameters				Bioactive Compounds	Bioactivity	Extraction Yield (%)	Reference
	Solvent (*v*/*v*)	Temperature (°C)	Pressure (MPa)	Extraction Time (min)				
*S. muticum*	EtOH:Water (75:25)	120	10.3	20	Phlorotannins	Antioxidant	40.1 ± 0.7	[30]
*S. japonica*	Ionic liquids	100–250	50 *	5	Phenolics	Antioxidant	---	[31]
*F. vesiculosus* *C. tomentosum*	Water (100 mL/min)	Room–90	100 *	100	Phlorotannins Phenolics Melanoidins	Neuroprotection Antioxidant	20.5	[34]
							4.1	
		90–140		90			56.0	
							7.0	
		140–190		90			65.9	
							33.6	
		190–250		100			73.7	
							51.4	
*F. serratus* *L. digitata* *G. gracilis* *C. fragile*	EtOH:Water (80:20)	100	6.9	25	Polyphenols	---	31.7	[38]
							24.8	
							21.8	
							26.9	
	Methanol:Water (70:30)	90	6.9	25			29.2	
							28.2	
							24.5	
							24.6	
*H. elongata* *E. bicyclis*	Water (100 mL/min)	Room–90	100 *	100	Phlorotannins Phenolics Melanoidins	Antioxidant Scavenging activity,	---	[75]
		90–140		90				
		140–190		90				
		190–250		100				
*H. elongata*	EtOH	50	10.3	20	Fucoxanthin	Antioxidant	8.29	[76]
		100					10.56	
		150					19.23	
		200					36.91	
	Hexane	50					3.41	
		100					3.50	
		150					4.72	
		200					7.59	
	H_2_O	50					9.51	
		100					15.08	
		150					46.43	
		200					51.56	
*F. vesiculosus*	Hexane	80	10	10	Fatty acids (oleic acid, ARA, EPA)	Antioxidant, antibacterial	2.79 ± 0.12	[77]
		120					3.72 ± 0.24	
		160					4.49 ± 1.54	
	EtOH	80					4.72 ± 0.11	
		120					5.59 ± 0.21	
		160					7.03 ± 1.79	
	Ethyl acetate	80					9.01 ± 1.24	
		120					10.73 ± 0.23	
		160					12.90 ± 1.2	
	Acetone	80					8.85 ± 1.51	
		120					11.98 ± 1.18	
		160					12.89 ± 0.68	
	EtOH:Water (50:50)	80					34.85 ± 3.11	
		120					41.49 ± 1.47	
		160					57.19 ± 2.38	
*K. alvarezii*	Ionic liquids	60–180	5	30–40	ĸ-carrageenan	Antioxidant	Up to 78.75	[78]
*S. japonica*	DES	60–160	5–60 *	10–25	Alginate	Antioxidant	5.37–27.21	[79]
					Fucoidan		5.08–15.70	

* bar.

**Table 6:** Ultrasound-Assisted Extraction for bioactive compounds from macroalgae, extraction parameters, bioactive compounds targeted, and extraction yield.

Macroalgae	Equipment	Extraction Parameters					Bioactive Compounds	Bioactivity	Extraction Yield (%)	Reference
		Solvent	Temperature (°C)	Frequency (kHz)	Power (W)	Extraction Time (min)				
*E. cava*	Bath	H_2_O	30	40	200	360	Polyphenols	Antioxidant	31.33 ± 1.3	[39]
						720			34.33 ± 1.4	
		Methanol (50%)				360			28.33 ± 1.6	
						720			30.67 ± 1.7	
		Methanol (100%)				360			16.00 ± 1.4	
						720			16.33 ± 1.8	
*H. banksii*	Bath	EtOH (70%)	30/40/50	50	150–250	20/40/60	Polyphenols	Antioxidant	---	[86]
*L. hyperborea* *A. Nodosum*	Probe	H_2_O	---	20	35.61 ^a^	15	Laminarin	Antioxidant	5.975 ± 0.467	[87]
									5.290 ± 0.480	
		0.1 M HCl							6.240 ± 0.008	
									5.822 ± 0.343	
*S. muticum*	Bath	H_2_O	50	50/60	400	60	Polyphenols	Prebiotic Glucosidase inhibition Antioxidant	25–27 ^b,c^	[88]
*O. pinnatifida*									45–47 ^b,c^	
*C. tomentosum*									47–49 ^b,c^	
*L. obtuse*	Bath	EtOH (95%)	30/35/40/45/50/55/60	40	250	30/40/50/60/70	Phenolic compounds Antioxidants	Antioxidant	---	[89]
*F. vesiculosus*	Bath	NADES		42	130	20 and 60	Phlorotannins, fucoxanthin, and ascorbic acid	Antioxidants	Phlorotannins–10–60 ^b,d^	[90]
									Fucoxanthin–0.2–0.4 ^b,d^	
									Ascorbic acid–0.05–0.4 ^b,d^	

a: intensity as W cm^−2^; b: Approximated values; c: Yield of polyphenols as g lyophilised extract/g dry seaweed; d: mg/g DW seaweed.

**Table 7:** Microwave- Assisted Extraction for bioactive compounds from macroalgae, extraction parameters, bioactive compounds targeted, and extraction yield.

Macroalgae	Extraction Parameters					Bioactive Compounds	Bioactivity	Extraction Yield (%)	Reference
	Solvent	Temperature (°C)	Solvent/Material Ratio (mL/g)	Power (W)	Extraction Time (min)				
*C. racemosa*	EtOH (40%, 50%, 60%)	30/40/50	20/30/40	100/200/300	30/40/50	Polyphenols	Antioxidant	---	[100]
*E. prolifera*	EtOH 25%	---	20	450	20	Polyphenols	---	0.655 ± 0.036 ^a^	[101]
	EtOH 30%		25		25			0.864 ± 0.017 ^a^	
	EtOH 35%		30		30			0.889 ± 0.014 ^a^	
	EtOH 40%		35		35			0.877 ± 0.026 ^a^	
	EtOH 25%		30	500	20			0.893 ± 0.019 ^a^	
	EtOH 30%		35		25			0.906 ± 0.008 ^a^	
	EtOH 35%		20		30			0.850 ± 0.042 ^a^	
	EtOH 40%		25		35			0.742 ± 0.028 ^a^	
	EtOH 25%		35	550	20			0.825 ± 0.015 ^a^	
	EtOH 30%		30		25			0.708 ± 0.034 ^a^	
	EtOH 35%		25		30			0.894 ± 0.016 ^a^	
	EtOH 40%		20		35			0.887 ± 0.038 ^a^	
	EtOH 25%		25	600	20			0.874 ± 0.027 ^a^	
	EtOH 30%		20		25			0.887 ± 0.017 ^a^	
	EtOH 35%		35		30			0.643 ± 0.019 ^a^	
	EtOH 40%		30		35			0.792 ± 0.043 ^a^	
*C. flexuosum*	H_2_O	135/160/185	---	---	1/3/5/10/15/20	Polyphenols	---	---	[102]
	Acetone								
	EtOH								
	Propan-1-ol								
	Ethyl acetate								
*U. prolifera*	0.1 M HCl	90	---	500	15	Polysaccharides	Antioxidant Anti-hyperlipidemic	18.0–20.0 ^b^	[103]
		120						6.0–8.0 ^b^	
		150						6.09 ± 0.65	
	0.01 M HCl	90						31.0–33.0 ^b^	
		120						36.38 ± 0.94	
		150						28.0–30.0 ^b^	
	0.05 M HCl	90						27.0–29.0 ^b^	
		120						18.0–20.0 ^b^	
		150						6.0–8.0 ^b^	
*C. flexuosum*	H_2_O	160	30	---	3	Phlorotannins	Antioxidant	15.8 ± 0.3	[104]
*C. plumosum*								9.2 ± 0.6	
*E. radiata*								2.0 ± 0.1	
*F. vesiculosus*	NADES	100–200	10–100 ^c^	---	10–60	Polysaccharides	Antioxidant	91.02–115.44 ^d^	[105]

a: Yield of polyphenols as mg GAE/g seaweed on a dry weight basis; b: Approximated values; c: mg/mL; d: mg/g.

**Table 8:** Enzyme-Assisted Extraction for bioactive compounds from macroalgae, extraction parameters, bioactive compounds targeted, and extraction yield.

Macroalgae	Extraction Parameters					Bioactive Compounds	Bioactivity	Extraction Yield (%)	Reference
	Solvent/Buffer	Temperature (°C)	pH	Extraction Time (h)	Enzymes				
*S. muticum*	0.1 M Phosphate buffer	50	7.0	2	Alcalase	Polyphenols	Antioxidant	13.6 ± 1.4	[30]
				4				17.81 ± 2.8	
	0.1 M Sodium acetate-acetic acid buffer	50	4.5	2	Viscozyme			20.6 ± 1.7	
				4				23.5 ± 0.1	
*C. fragile* * C. crispus **	Distilled water	50	---	3	Cellulase *	Proteins	Anti-viral	2.6 ± 0.2	[115]
						Neutral Sugars		4.3 ± 0.2	
						Uronic Acid		0.23 ± 0.0	
						Sulphates		0.5 ± 0.0	
					Neutrase *	Proteins		2.9 ± 0.1	
						Neutral Sugars		5.4 ± 0.3	
						Uronic Acid		0.1 ± 0.0	
						Sulphates		0.5 ± 0.0	
					Cellulase **	Proteins		---	
						Neutral Sugars		7.1 ± 0.3	
						Uronic Acid		1.2 ± 0.0	
						Sulphates		11.7 ± 0.1	
					Neutrase **	Proteins		---	
						Neutral Sugars		---	
						Uronic Acid		0.8 ± 0.0	
						Sulphates		8.4 ± 0.1	
					β-glucanase **	Proteins		4.1 ± 0.4	
						Neutral Sugars		9.3 ± 0.2	
						Uronic Acid		1.0 ± 0.0	
						Sulphates		8.0 ± 0.1	
					Ultraflo **	Proteins		5.8 ± 0.5	
						Neutral Sugars		21.9 ± 0.4	
						Uronic Acid		1.4 ± 0.0	
						Sulphates		11.0 ± 0.1	
*U. pinnatifida*	---	37	6.2	2	Alginate lyase	Fucoxanthin Lipids	---	---	[116]
*E. cava* *I. okamurae* *S. fullvelum* *S. horneri* *S. coreanum* *S. thunbergii* *S. lomentaria*	0.2 M PB ^b^	40	6.0	12	Protamex	Polyphenols	Antioxidant	---	[117]
	0.2 M PB	40	6.0	12	Kojizyme				
	0.2 M PB	50	6.0	12	Neutrase				
	0.2 M PB	50	7.0	12	Flavourzyme				
	0.2 M PB	50	8.0	12	Alcalase				
	0.1 N AB	50	4.5	12	Viscozyme L				
	0.1 N AB	50	4.5	12	Celluclast				
	0.1 N AB ^a^	60	4.5	12	AMG				
	0.2 M PB	60	6.0	12	Termamyl				
	0.2 M PB	60	7.0	12	Ultraflo				
*S. polycystum* *P. commersonii* *A. Pygmaea* *G. corticata* *C. herpestica* *C. antennina* *G. lithophila* *C. minima* *U. fasciata* *S. natans*	Deionised water	50	4.5	24	Viscozyme	Carbohydrates Proteins Polyphenols	Antioxidant Anti-inflammatory	See Table 9	[118]
		50	4.5	24	Celluclast				
		60	4.5	24	Termamyl				
		60	6	24	AMG				
		0	7	24	Ultraflo				

*: Enzymes applied to the macroalgae *C. crispus*; **: Enzymes applied to the macroalgae *C. fragile*; a: Acetate buffer; b: Phosphate buffer.

**Table 9:** Enzyme-Assisted Extraction for bioactive compounds from various macroalgae using different enzymes and respective extraction yields (adapted from [118]).

Extraction Yield [%]										
Enzyme	*S. polycystum*	*S. natans*	*P. commersonii*	*C. minima*	*C. herpestica*	*C. antennina*	*U. fasciata*	*A. pygmaea*	*G. corticata*	*G. lithophila*
Viscoenzyme	12.5 ± 1.3	21.0 ± 1.0	21.5 ± 0.9	9.0 ± 1.0	14.5 ± 0.6	33.5 ± 2.0	25.0 ± 1.8	17.0 ± 0.5	33.5 ± 1.7	36.0 ± 1.1
Celluclast	15.0 ± 0.9	23.5 ± 0.9	26.0 ± 1.2	12.0 ± 0.8	17.5 ± 0.9	39.5 ± 0.8	27.0 ± 0.9	17.5 ± 0.6	36.0 ± 0.8	40.0 ± 0.9
AMG	14.5 ± 0.5	22.5 ± 0.9	23.5 ± 0.9	8.5 ± 0.8	14.5 ± 0.7	35.5 ± 1.3	25.5 ± 0.8	16.5 ± 0.9	34.5 ± 0.7	36.0 ± 1.1
Termamyl	13.0 ± 0.9	22.0 ± 1.1	22.5 ± 0.8	8.0 ± 0.7	14.0 ± 0.6	34.5 ± 0.8	26.0 ± 1.2	16.5 ± 0.9	34.0 ± 0.3	37.0 ± 0.6
Ultraflo	10.5 ± 0.2	21.5 ± 0.4	17.5 ± 0.2	6.5 ± 0.7	15.0 ± 0.2	37.5 ± 0.6	24.0 ± 0.5	12.0 ± 0.9	29.5 ± 0.7	34.0 ± 0.2

**Table 10:** Ultrasonic-Microwave-Assisted Extraction for bioactive compounds from macroalgae, extraction parameters, bioactive compounds targeted, and extraction yield.

Macroalgae	Extraction Parameters					Bioactive Compounds	Bioactivity	Extraction Yield (%)	Reference
	Temperature (°C)	Ultrasonic Power (W)	Ultrasonic Amplitude (%)	Microwave Power (W)	Extraction Time (min)				
*A. nodosum*	36.2–98	500	20	250	2/5	Carbohydrates Polyphenols	Antioxidant	---	[111]
			50	600					
			100	1000					
*P. haitanensis*	70	50	---	500	20	Polysaccharides	---	12.80	[122]
					30			12.65	
					40			12.70	
	80				20			11.80	
					30			21.10	
					40			10.45	
	90				20			9.50	
					30			15.25	
					40			10.75	

**Table 11:** Liquefied Gas Extraction for bioactive compounds from macroalgae, extraction parameters and bioactive compounds targeted.

Macroalgae	Extraction Parameters				Bioactive Compounds	Extraction Yield (%)	Reference
	Solvent	Temp (°C)	Pressure (MPa)	Extraction Time (min)			
*U. pinnatifida*	DME (286 g)	25	0.59	43	Fucoxanthin	390 ^a^	[124]
*M. nitidum*	DME (216 g)	25	0.79	33	Lutein	0.30 ^b^	[127]
					Lipids	3.28	

a: μg/g DW; b: mg/g DW.

**Table 12:** Advantages and disadvantages of the alternative and conventional extraction techniques, and respective compounds extracted.

Extraction Technique	Compounds Extracted	Advantages	Disadvantages
SLE	Polyphenols	High yields of extraction; allows the extraction of thermolabile compounds; cheap technique, simple method; low temperatures.	Use of organic solvents; not eco-friendly; only polar compounds can be extracted; long extraction time.
	Fucoxanthin		
	Polysaccharides		
Soxhlet	Lipids	Simple methodology; Can use larger sample mass; Cheap technique.	Long time extraction; Large amounts of solvents; High energy consumption; Concentration steps are required; Analytes may decompose thermally.
SFE	Fucoxanthin	Extraction of important compounds; Possibility of having extracts already dried; Low quantities of solvent; High speed of analysis.	Low extraction yields; Expensive; Only extracts non-polar solvents.
	Phlorotannins		
	Carotenoids		
PLE	Polyphenols	Less solvent, Short extraction time; Easy operation.	Not suitable for thermolabile compounds; High instrument cost.
	Fucoxanthin		
	Fatty acids		
UAE	Polyphenols	High extraction efficiency; Possibility to be up-scaled; Reduced extraction time; Good for thermolabile compounds.	Decline of power with time; High capital cost.
MAE	Polyphenols	Reduced solvent usage; Possibility to be up-scaled; Higher extraction rate; Improved extraction yield: Simple instrumentation.	Not suitable for thermolabile compounds; High capital cost.
	Polysaccharides		
	Phlorotannins		
EAE	Polyphenols	Can be solvent-free; Possibility to be up-scaled; Possibility to be up-scaled; Eco-friendly; Higher extraction yields.	Slow process; Difficulty to maintain optimum treatment time and temperature conditions.
	Proteins		
	Neutral Sugars		
	Uronic Acid		
	Sulfates		
	Carbohydrates		
	Fucoxanthin		
UMAE	Polysaccharides	Higher extraction efficiency; Shorter extraction time; Reduced by-products.	Higher instrument cost.
	Carbohydrates		
	Polyphenols		
LGE	Carotenoids	Avoids sample drying, cell disruption, and solvent evaporation; Simple, cheaper and low energy consumption system; Replacement of toxic solvents for lipophilic compounds.	Isobaric mode: Expensive equipment; Frequent maintenance operations; The size of compressors is a limiting factor for large industrial applications. Non-isobaric mode: Careful design and monitoring of boiler and condenser
	Lipids		
